# Context-dependent variability in the predicted daily energetic costs of disturbance for blue whales

**DOI:** 10.1093/conphys/coaa137

**Published:** 2021-01-16

**Authors:** Enrico Pirotta, Cormac G Booth, David E Cade, John Calambokidis, Daniel P Costa, James A Fahlbusch, Ari S Friedlaender, Jeremy A Goldbogen, John Harwood, Elliott L Hazen, Leslie New, Brandon L Southall

**Affiliations:** 1 Department of Mathematics and Statistics, Washington State University, Vancouver, WA 98686, USA; 2 School of Biological, Earth and Environmental Sciences, University College Cork, Cork T23 N73K, Ireland; 3 SMRU Consulting, Scottish Oceans Institute, University of St Andrews, St Andrews KY16 8LB, UK; 4 Department of Biology, Hopkins Marine Station, Stanford University, Pacific Grove, CA 93950, USA; 5 Cascadia Research Collective, Olympia, WA 98501, USA; 6 Department of Ecology and Evolutionary Biology, University of California, Santa Cruz, CA 95060, USA; 7 Southall Environmental Associates, Inc., Aptos, CA 95003, USA; 8 Centre for Research into Ecological and Environmental Modelling, University of St Andrews, St Andrews KY16 9LZ, UK; 9 Southwest Fisheries Science Center, Environmental Research Division, National Oceanic and Atmospheric Administration (NOAA), Monterey, CA 93940, USA; 10 Institute of Marine Sciences, University of California, Santa Cruz, CA 95064, USA

**Keywords:** Behavioral response studies, data integration, energy budget, marine mammals, navy sonar, population consequences of disturbance

## Abstract

Assessing the long-term consequences of sub-lethal anthropogenic disturbance on wildlife populations requires integrating data on fine-scale individual behavior and physiology into spatially and temporally broader, population-level inference. A typical behavioral response to disturbance is the cessation of foraging, which can be translated into a common metric of energetic cost. However, this necessitates detailed empirical information on baseline movements, activity budgets, feeding rates and energy intake, as well as the probability of an individual responding to the disturbance-inducing stressor within different exposure contexts. Here, we integrated data from blue whales (*Balaenoptera musculus*) experimentally exposed to military active sonar signals with fine-scale measurements of baseline behavior over multiple days or weeks obtained from accelerometry loggers, telemetry tracking and prey sampling. Specifically, we developed daily simulations of movement, feeding behavior and exposure to localized sonar events of increasing duration and intensity and predicted the effects of this disturbance source on the daily energy intake of an individual. Activity budgets and movements were highly variable in space and time and among individuals, resulting in large variability in predicted energetic intake and costs. In half of our simulations, an individual’s energy intake was unaffected by the simulated source. However, some individuals lost their entire daily energy intake under brief or weak exposure scenarios. Given this large variation, population-level models will have to assess the consequences of the entire distribution of energetic costs, rather than only consider single summary statistics. The shape of the exposure-response functions also strongly influenced predictions, reinforcing the need for contextually explicit experiments and improved mechanistic understanding of the processes driving behavioral and physiological responses to disturbance. This study presents a robust approach for integrating different types of empirical information to assess the effects of disturbance at spatio-temporal and ecological scales that are relevant to management and conservation.

## Teaser

Management and conservation require assessments of the population-level effects of short-term individual responses to disturbance. To bridge this gap, we integrate diverse data on blue whales and convert responses to sonar into corresponding energetic costs. Due to behavioral variation, predicted costs were extremely variable in space, time and among individuals.

## Introduction

Exposure to human activities can cause changes in the behavior and physiology of individual animals (Frid and Dill, 2002; Beale and Monaghan, 2004). These responses need to be understood in the context of their long-term effects on individual vital rates (such as survival or reproduction) and, ultimately, population dynamics in order to most effectively inform management actions (Gill *et al.,* 2001; Pirotta *et al.,* 2018a; Ames *et al.,* 2020).

In the past two decades, concerns over the effects of military active sonar on marine mammals have stimulated an extensive empirical and analytical effort. This has included direct measurements of behavioral responses to sonar by means of Controlled Exposure Experiments (CEEs) (Southall *et al.,* 2016; Harris *et al.,* 2018). Most CEEs use animal-borne electronic loggers and return detailed, high spatio-temporal resolution (meters, seconds) information on 3D individual movements following exposure, e.g. changes in diving behavior, orientation, vocalizations and location (e.g. Goldbogen *et al.,* 2013; Friedlaender *et al.,* 2016; Southall *et al.,* 2019a). Results are typically synthesized into functions describing the relationship between a given level of exposure (e.g. received levels of sonar) and the probability of response (Harris *et al.,* 2018). Parallel analytical developments have formalized a suitable framework to model long-term, population-level effects of these short-term responses (Pirotta *et al.,* 2018a). In contrast to CEEs, this framework operates over broader spatio-temporal scales (e.g. tens of km, days), in part due to the computational and empirical limitations associated with modelling the entire lifetime of multiple individuals from long-lived, wide-ranging species (e.g. Villegas-Amtmann *et al.,* 2017; Nabe-Nielsen *et al.,* 2018; Hin *et al.,* 2019; Pirotta *et al.,* 2019). Therefore, it has proven challenging to integrate the detailed empirical information provided by CEEs into a population-level model.

Cascading effects from behavior to vital rates are mediated by an alteration of each individual’s overall health status, for example via disruption of energy budgets (National Academies, 2017; Pirotta *et al.,* 2018a). For cetaceans, this disruption is mostly driven by an interruption of feeding activity (Noren *et al.,* 2016). Energy can act as a common currency to link the short-term costs of fine-scale behavioral changes observed during CEEs to the longer-term effects on survival and reproduction (Houston and McNamara, 2014), thereby facilitating the integration of these responses into population-level models.

The quantification of individual energy budgets requires empirical information on the patterns of behavior animals exhibit in the absence of disturbance, such as the time they allocate to different activities within a day (i.e. their activity budget), the rates at which they feed and their movements within an area of interest (e.g. Boyd, 1999; Hamel and Côté, 2008; Louzao *et al.,* 2014). Moreover, data on the prey they are targeting (such as its patterns of availability, density and distribution) are essential in order to quantify energy intake, the energetic efficiency of foraging and the opportunity to compensate for interrupted feeding (Bowen *et al.,* 2002; Grémillet *et al.,* 2004; Goldbogen *et al.,* 2019; Booth, 2020; Friedlaender *et al.,* 2020). These data are increasingly available as technology improves our ability to monitor animals’ activity over extended periods and to sample the environment in which they move (Cade and Benoit-Bird, 2014; Hazen *et al.,* 2015; Calambokidis *et al.,* 2019; Irvine *et al.,* 2019). However, behavior, body condition, reproductive state, home range and resource availability will vary in space and time and among individuals, resulting in large differences in individual energy budgets.

Changes in behavior resulting from exposure to a disturbance-inducing stressor can also depend on context (e.g. Ellison *et al.,* 2012). Internal factors (such as behavioral state, body condition or previous experience), spatial relationships of source and receiver and features of the surrounding environment (e.g. prey quality) can affect the probability of an animal altering its behavior (Ellison *et al.,* 2012; Houser *et al.,* 2013; Friedlaender *et al.,* 2016; DeRuiter *et al.,* 2017; Southall *et al.,* 2019a). Due to the logistical difficulties and high associated costs of CEEs, data from these experiments often do not support the estimation of exposure-response (ER) functions based on separate combinations of environmental or behavioral conditions (Southall *et al.,* 2016).

In this study, we integrated diverse data sources to predict the daily energetic costs of simulated disturbance scenarios on individual blue whales (*Balaenoptera musculus*) from the Eastern North-Pacific (ENP) population. Because its range overlaps with an area used by the US Navy for military training and testing exercises (Calambokidis *et al.,* 2009), this population has been the subject of a large behavioral response study, which has generated an extensive CEE dataset (Southall *et al.,* 2016, 2019a). Data from experimental exposures were used to build state- and range-specific discrete ER functions, as well as continuous functions for noise received level (RL) and range from the source. Moreover, we derived unprecedented information on baseline behavioral states, activity budgets, feeding rates, feeding bouts and movements over multiple days or weeks from the activity patterns of individuals instrumented with electronic loggers and that were not experimentally exposed to sonar (Szesciorka *et al.,* 2016; Calambokidis *et al.,* 2019). Finally, we included measurements of krill densities collected around feeding whales (Goldbogen *et al.,* 2015, 2019; Hazen *et al.,* 2015) to quantify expected energy intake in undisturbed conditions. These data sources were combined to develop daily simulations of whale movement, feeding behavior and exposure to localized noise sources of increasing duration and intensity. A bioenergetic model (Pirotta *et al.,* 2018b, 2019) was used to estimate individual daily net energy intake in disturbed and undisturbed conditions, which provides the common metric needed for the integration of experimental results into models of population-level consequences. We also assessed the variation in predicted costs as a function of the ER curve used, the spatio-temporal subset of activity data that was used to inform whale behavior, krill density distribution and whale size, and identified the most important data gaps.

## Materials and methods

### Multi-day tag data collection and analysis

**Figure 1:**
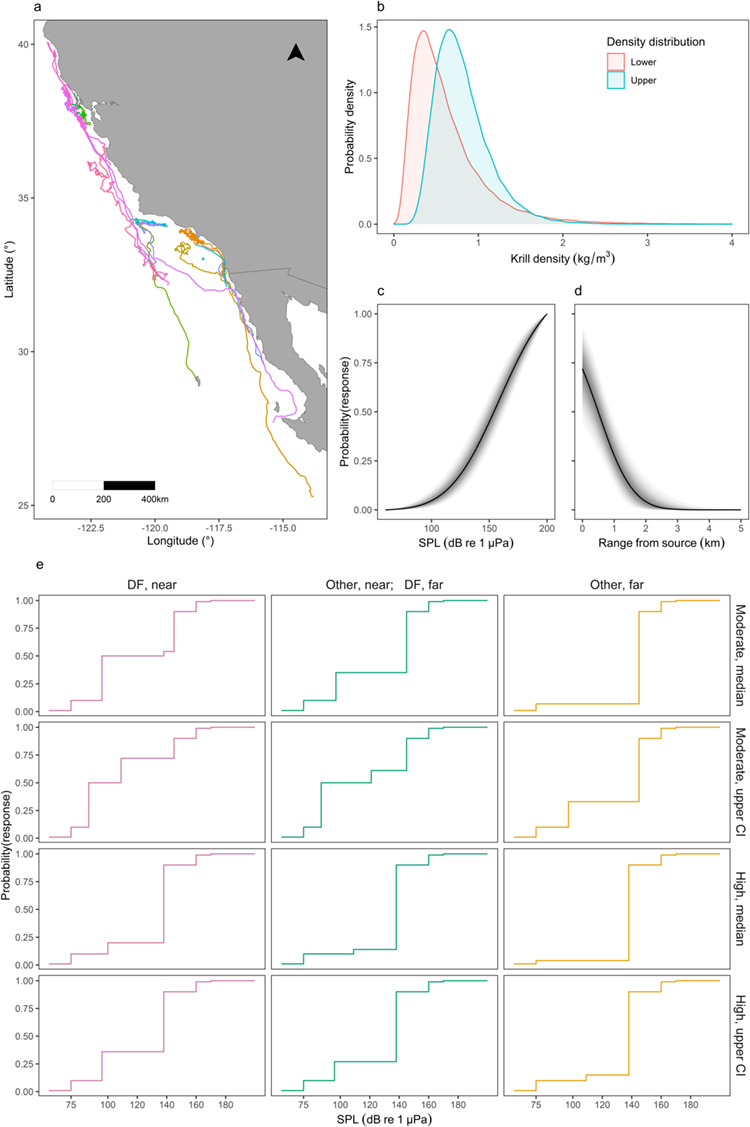
Data used to inform disturbance simulations. (a) Map of multi-day tag data, colored by deployment. (b) Pooled lower and upper krill density distributions in California. Posterior exposure-response (ER) function for (c) SPL and (d) range from source. The solid line represents the median, while shaded areas represent 95% credible intervals. (e) Discrete ER functions derived from key assumptions and the survival analysis in Southall *et al.* (2019a) for individuals in deep-feeding (DF) or non-deep-feeding (Other) state, either near (within 1 km) or far from (beyond 1 km) the source (columns). Functions were derived from the median and upper confidence interval (CI) of the moderate and high response severity curves (rows).

Between 2014 and 2019, individual blue whales were instrumented with Wildlife Computers TDR10-F tags (*n* = 21) and Acousonde acoustic tags (*n* = 6), returning GPS location, depth and, in most configurations, 3D accelerometry data for an average of 8 d (range, 1–32 d; Fig. 1a). Details of tag configurations, deployment and field procedures are provided in Supplementary Methods S1 and in Szesciorka *et al.* (2016) and Calambokidis *et al.* (2019). Raw tag data were processed following Cade *et al.* (2016) to identify feeding events, or lunges. Data were summarized into hourly locations, number of detected feeding lunges (representing hourly lunge rates) and mean lunge depth, as detailed in Supplementary Methods S1, where we also discuss differences between tags with and without 3D accelerometry sensors. The hourly scale was used to match the resolution of the simulations (see below), and represented a trade-off between retaining sufficient detail of an individual’s behavioral variation while keeping the simulations tractable.

An individual was assumed to be in a feeding state in any hour in which at least one lunge was detected (Goldbogen *et al.,* 2012). For simplicity, we did not distinguish among hours based on the proportion of time spent lunging. Blue whales in this population engage in two distinct feeding modes, deep and shallow lunge feeding (Goldbogen *et al.,* 2015), which involve a different number of lunges per dive and trade-off between oxygen access at the surface and food resources at depth (Hazen *et al.,* 2015). Mean lunge depth in each hour was used to determine the predominant feeding mode for that hour (shallow or deep feeding), using a Gaussian mixture model with two components, fitted with package mixtools version 1.1.0 (Benaglia *et al.,* 2009). The first component had a mean of 33 m (SD = 20 m), corresponding to shallow feeding, while the second component was centered on 157 m (SD = 75 m), representing deep feeding. In addition, we extracted the temporal gaps (in hours) between foraging bouts, that is, the duration of any break (≥1 h) in a sequence of consecutive hours spent in either feeding state (excluding any non-foraging time at the start and at the end of each deployment).

We used a Markov chain algorithm (package markovchain version 0.6.9.7; Spedicato, 2017) to estimate the transition probabilities between hourly states (deep feeding, shallow feeding and not feeding) and the corresponding stationary distribution (which represents the expected proportion of time spent in each state, i.e. a whale’s daily activity budget). Daily activity budgets could vary in space and time. Although available data could not support a full exploration of spatio-temporal behavioral differences, we wanted to verify whether such variation existed. Therefore, in addition to including the full dataset, we repeated the analysis using only data collected in specific portions of the range or times of the year. Specifically, we defined four subsets of the data, corresponding to the two spatial and the two temporal subsets containing the highest number of complete individual days (i.e. days with 24 hourly records for a given individual). Temporal subsets were defined based on month, while spatial subsets were defined using the latitudinal ranges used to model individual movement in Pirotta *et al.* (2018b, 2019), to facilitate future integration of these results in a population-level model. As a result, the two spatial subsets included data in the latitudinal ranges 33.8°N--34.4°N and 37.6°N--38.4°N, and the two temporal subsets corresponded to data from July and October.

We used the Minimum Convex Polygon estimator in package adehabitatHR version 0.4.16 (Calenge, 2006) to estimate the area over which an individual ranged in each day (hereafter, area covered per day). This estimation was limited to complete individual days and days where there was some feeding, i.e. where at least one hour contained feeding lunges.

### CEEs

Between June and October 2010 to 2014, 42 individual blue whales were tagged with archival electronic loggers in the Southern California Bight and exposed to experimental and operational sound sources. Details of the experimental design, field protocols and permits are provided in Southall *et al.* (2012, 2016, 2019a). The tags recorded fine-scale, 3D movements, which were analyzed using change-point methods and a standardized expert-scoring procedure to determine the occurrence, time and severity of any behavioral change following exposure, as described in detail in Miller *et al.* (2012) and Southall *et al.* (2019a).

### Development of ER probability functions

Using the CEE data, we developed three types of ER functions (state- and range-specific discrete functions, a continuous function for noise RL and a continuous function for range from the source) and compared the resulting energetic costs (see below), to investigate the influence of context-dependency and of the metric used to represent the stressor.

Southall *et al.* (2019a) applied recurrent event survival analysis (Harris *et al.,* 2015) to the results of the CEEs to derive blue whale response probability as a function of exposure level in different exposure contexts (differentiated by behavioral state and the range from the source to the whale), for moderate and high response severity scores. Responses were strongly context-dependent but sample sizes for certain contexts were small or absent. First, we therefore derived relatively coarse ER functions from the results in Southall *et al.* (2019a). Distinct conditions were collapsed into three contexts, representing decreasing relative sensitivity: (i) deep-feeding, near (≤ 1 km from the source); (ii) deep-feeding, far (> 1 km from the source), and other states, near; and (iii) other states, far. Being in the most sensitive state (deep feeding) far from the source was therefore assumed to be comparable to being in another state but close to the source (Supplementary Methods S2, Fig. S4).

For each context, we related noise exposure level to a discrete set of response probabilities (1, 10, 50, 90, 99%) or defined the level at which response probability reached an asymptote. Across contexts, 1% response probability was defined as the estimated ambient noise in MFAS band (3–4 kHz) for sea state 3 conditions from Wenz (1962). Ten, 50 and 90% response probabilities were derived from the functions in Southall *et al.* (2019a) where possible. Where the corresponding curves reached an asymptote below these probability values, RLs were determined at respective asymptotes. Because all functions reached an asymptote below 90% probability, the RL corresponding to this probability was determined using estimates of effective quiet from humans, that is, 10 dB below estimates of temporary threshold shift (TTS), as proposed by Ward *et al.* (1976). TTS onset estimates for blue whales (as ‘low-frequency cetaceans’) were derived from Southall *et al.* (2019b). Further, these TTS onset estimates were used as the 99% response probability for all functions. This procedure returned four step functions (corresponding to the median and upper confidence interval of the survival analysis for moderate and high response severity scores) for each of the three exposure contexts, defining the probability of an individual in each state and range category responding to different RLs (Fig. 1e; Supplementary Methods S2).

In addition, we developed a continuous ER function for received root-mean-square (RMS) sound pressure level (hereafter, SPL), pooling the data from all individuals irrespective of behavioral state and range from the source. We used the expert scoring described in Southall *et al.* (2019a) to determine whether an individual was deemed to have responded with moderate or high response severity within each experimental exposure, and extracted SPL at the time of the identified behavioral change. If individuals did not respond, we extracted the maximum SPL received during the experiment. The data were then analyzed using the Bayesian approach described in Miller *et al.* (2014) (Supplementary Methods S2). Finally, we developed a second continuous function, where range from the noise source was used as the exposure term. We extracted the distance (in km) between an individual and the source at the time of an identified response or, for individuals that did not respond, the minimum distance reached during the experiment, and analyzed these data using a modified version of the approach in Miller *et al.* (2014) (Supplementary Methods S2).

### Krill density data

Acoustic backscatter data targeting krill were collected using Simrad EK60 or EK80 transceivers at 38 and 120 kHz in the Southern California Bight and in Monterey Bay between 2011 and 2018, following the field protocols described in Goldbogen *et al.* (2019). Hydroacoustic data were analyzed according to the predator-scale method described in Cade *et al.* (In press) and Cade (2019). The method generates krill density distributions that represent how acoustic cells the size of an average whale engulfment are distributed within cells the size of an average whale’s horizontal and vertical movement during a feeding dive. Two lognormal density distributions were calculated for each sampling location: one corresponding to the mean distribution, assuming a randomly foraging whale (hereafter lower extreme), and the other using the top 50% of data in each dive-sized cell (hereafter upper extreme), assuming that a whale chooses where to forage in a patch to maximize efficiency (Cade *et al.*, In press; Cade, 2019; Goldbogen *et al.,* 2019). The means and standard deviations of the distributions at each location were then pooled to obtain two distributions of krill density for the broader California region (lower and upper extremes), which are representative of krill biomass available to blue whales at a spatial scale matching their foraging behavior (Cade *et al.*, In press). We investigated differences in krill density in shallow and deep patches (*sensu*  Goldbogen *et al.,* 2015), but did not find any, possibly due to sampling limitations (e.g. the small number of shallow samples, or the difficulty of characterizing shallow patches using a downward echosounder). Therefore, the same distributions were used for all depths.

### Simulations

**Figure 2:**
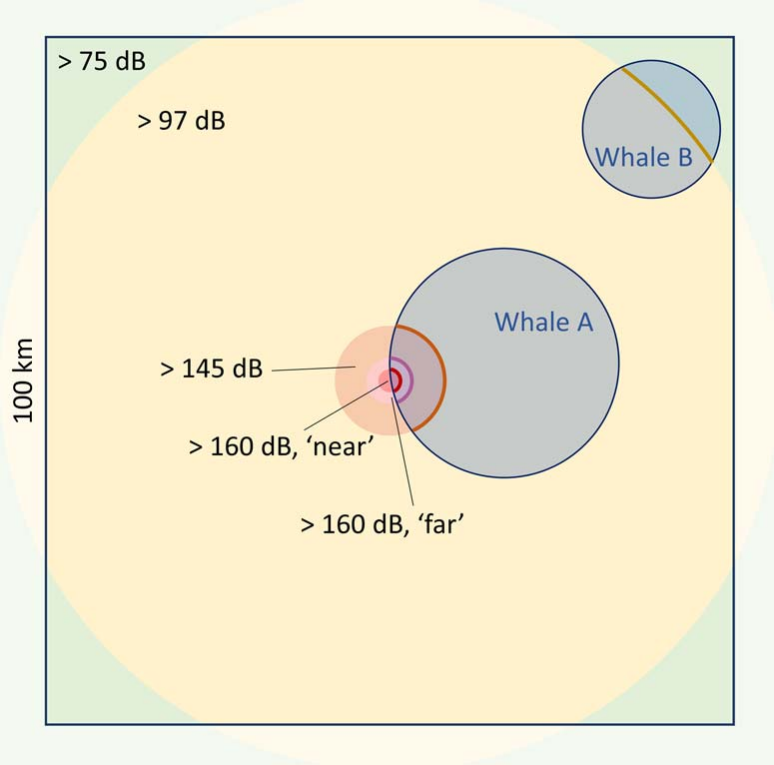
Schematic representation of the simulations using one of the discrete exposure-response functions. Shaded blue circles provide examples of the areas covered per day by individual whales, resulting in differential overlap with exposed areas. The other shaded regions represent the areas exposed to different ranges of received levels, where response probability corresponds to the probability at the upper extreme of that range (warmer colors indicate higher received levels). Relative ranges are not to scale.

We developed daily simulations of whale behavior to estimate the energetic costs of a disturbance-inducing event of increasing duration and intensity, occurring at a fixed position within a 100 km × 100 km rectangle (chosen to match the spatial resolution of the model used by Pirotta *et al.* (2018b, 2019); Fig. 2). Given source level (SL), a simple, spherical noise propagation model was used to determine the ranges at which RLs of interest were reached (Au and Hastings, 2008). For each replicate, we sampled a random day from the activity data and calculated the proportional overlap between the area covered on that day and the area between two defined RLs of interest, taken to represent the proportion of a day an individual was exposed to RLs in that range.

For the discrete ER functions, an individual could respond every 6 minutes, matching the assumptions underpinning their derivation (Supplementary Methods S2). The probability of responding at each interval was independent of any previous exposure or response to sonar. Six-minute intervals over a day were thus randomly assigned to portions of the covered area exposed to different RL ranges, based on the proportional overlaps, and a behavioral state, based on the activity budget on the sampled day. Given the time and duration of the simulated source, we determined if each interval was exposed and the corresponding range of RL experienced. Response probability was then determined from an ER function. Different functions were used depending on the feeding state in each interval and whether the individual was within 1 km of the source (‘near’) or further away (‘far’).

Using the continuous ER functions, an individual could respond every 30 min, i.e. the typical experimental exposure duration in Southall *et al.* (2019a). This temporal scale was thus used to match the temporal scale underlying the development of the continuous ER functions. For SPL, we used bins of 10 dB and calculated the expected range at which those RL were reached. For range from source, we considered six distance bins (0–1,..., 4–5, > 5 km) and calculated the proportion of a day an individual spent in each bin as the proportional overlap between the area covered on that day and the area in that distance bin. In both cases, response probabilities associated with each bin were sampled from a truncated normal distribution defined by the posterior mean and standard deviation of the estimated ER curve. For both the discrete and continuous functions, we conservatively assumed that a foraging individual within the area exposed to a given bin of RL or range from source responded with a probability corresponding to the upper extreme of that bin (Fig. 1e; Supplementary Methods S3).

For each potential response, we randomly sampled an empirical gap between foraging bouts from the tag data, providing a likely inflated (and thus appropriately precautionary given the underlying uncertainty) estimate of the time required to find a new krill patch after disturbance. All time intervals corresponding to a behavioral response followed by a gap were considered lost. However, if an animal had responded in a previous interval and was still looking for a new patch, it did not respond again or lose additional time. For each day, we then tallied the total number of deep-feeding and shallow-feeding hours lost, representing the total feeding time loss.

Using the state-specific hourly lunge rates on that day, we computed the total number of lunges lost. For each lost lunge, we drew a value of krill density from the pooled lognormal krill density distribution. Given the length of the simulated individual (affecting buccal size, as per Pirotta *et al.,* 2018b, 2019), krill energy density in the California Current (Chenoweth, 2018) and assimilation efficiency (Lockyer, 1981), lost lunges were translated into total (gross) energy lost on a day, representing the daily energetic cost of that disturbance scenario.

We estimated the theoretical gross energy acquired on that day without disturbance, given the bioenergetics equations in Pirotta *et al.* (2018b, 2019) (Supplementary Methods S3). We divided the total energy loss by the gross energy acquired in undisturbed conditions and obtained the proportional loss in energy acquired. This metric could vary between 0 and 1 and summarized the proportion of energy acquisition that was lost following disturbance.

The bioenergetics equations in Pirotta *et al.* (2018b, 2019) were also used to estimate daily energy expenditure, given the activity budget on each day (Supplementary Methods S3). Net energy intake was then computed as acquired minus expended energy for undisturbed and disturbed conditions, adjusting energy expenditure following disturbance in light of the altered activity budget. Whenever simulated krill densities resulted in foraging costs exceeding energy acquired, we set the net intake from foraging to 0; however, the daily net intake could still be negative if maintenance costs exceeded foraging gains. The daily costs of reproduction that females could potentially incur were calculated for an individual in the middle of pregnancy (for gestation), or assuming an individual delivered the maximum daily amount of milk to the calf (for lactation) (Pirotta *et al.,* 2018b).

Simulations were repeated for: 1) five SL (235 dB re 1 μPa, i.e. the nominal intensity of 53C sonar; 210 dB re 1 μPa, i.e. the highest SL achieved during CEEs, comparable to the intensity of other Navy MFAS systems, including helicopter-dipping (AN/AQS-22) sonar; 200, 180 and 160 dB re 1 μPa, covering the full range of transmitted source levels associated with the wide variety of military activities occurring in the study area); 2) seven durations of the disturbance-inducing event (6 min, i.e. the average duration of 10 sonar pings at maximum level; 30 min, i.e. the average CEE duration; 60, 120, 360, 720 and 1440 min); 3) three source positions (at the center, in a corner or at the center of one side of the 100 km x 100 km rectangle); 4) three whale lengths (22, 25 and 27 m); 5) two krill density distributions (corresponding to the lower and upper extremes for the pooled distribution); 6) six ER functions (four discrete and two continuous). Each scenario resulting from the combination of these conditions was replicated 1000 times. Simulations were also repeated using only subsets of the multi-day tag data, corresponding to the two locations and the two months encompassing most data. For simplicity, simulation results are presented for a 22-m-long individual (the average asymptotic length of ENP blue whales; Gilpatrick and Perryman, 2008), feeding on krill densities drawn from the lower pooled distribution and assuming a discrete ER function corresponding to the median result of the survival analysis for moderate response severity scores. Results from the other simulated scenarios are discussed in comparison to these reference conditions.

Simulations were coded in R version 3.6.2 (R Core Team, 2019). The data and code to run these analyses are available via the Open Science Framework (https://osf.io/q5nbf/?view_only=f7bda77903594328a9ff30cc26b62b78). A list of all abbreviations is reported in Supplementary Methods S4.

## Results

After processing, the multi-day tags provided 5281 hourly records of activity and 134 complete days during which the animals engaged in some foraging activity (Fig. 1a). Deployments spanned from May to November, from Baja California Peninsula (Mexico) to northern California (25°N--40°N), but most data were collected between July and October, with animals concentrating in the Southern California Bight and in waters off San Francisco and Monterey. The area covered by individuals over the course of a day (with some foraging) varied between 12 and 1647 km^2^ (mean = 294 km^2^; standard deviation (SD) = 343 km^2^). The stationary distribution of the Markov chain suggested individuals spent a variable amount of time in different behavioral states. Different stationary distributions were also obtained when the algorithm was run on subsets of the data collected in the two most represented locations and months (Table 1). In contrast, state-specific lunge rates did not vary (Table 1; Supplementary Methods S3, Fig. S5). The temporal gaps between bouts of consecutive hours with feeding activity ranged between 1 and 226 h (mean = 10 h; SD = 20 h).

**Table 1:** Daily activity budget across data subsets

Data subset	% time not feeding	% time deep feeding	% time shallow feeding	Mean hours deep feeding [range]	Mean hours shallow feeding [range]	Median lunge rate deep feeding (SD); lunges/h	Median lunge rate shallow feeding (SD); lunges/h
Overall dataset	61	29	10	9 [0–17]	3 [0–18]	19 (8)	15 (10)
Latitude range 33.8°N--34.4°N	42	50	8	13 [0–17]	2 [0–11]		
Latitude range 37.6°N--38.4°N	46	29	25	7 [0–15]	6 [0–18]		
July	45	48	7	12 [0–17]	2 [0–9]		
October	65	32	3	9 [0–15]	1 [0–4]		

The lower and upper pooled krill density lognormal distributions taken to represent the broader California ecosystem had geometric means 0.513 kg/m^3^ and 0.757 kg/m^3^, and geometric SDs 1.917 kg/m^3^ and 1.468 kg/m^3^, respectively (Fig. 1b). The four discrete (median and upper confidence interval of the survival analysis for moderate and high response severity) and two continuous (for SPL and range from source) ER functions are represented in Fig. 1c-e. Gibbs Variable Selection excluded the effects of previous exposure and source type on response probability in the latter two functions.

**Figure 3:**
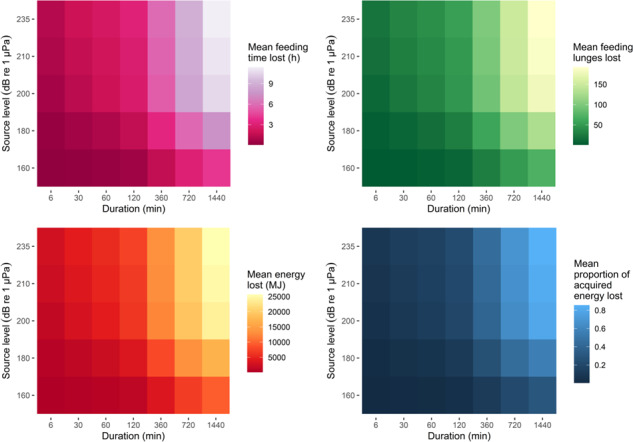
Predicted effects of disturbance for increasing source intensity and duration, under reference conditions (that is, assuming the discrete exposure-response function for median values under moderate response severity, the lower krill density distribution and a 22-m-long individual).

**Figure 4:**
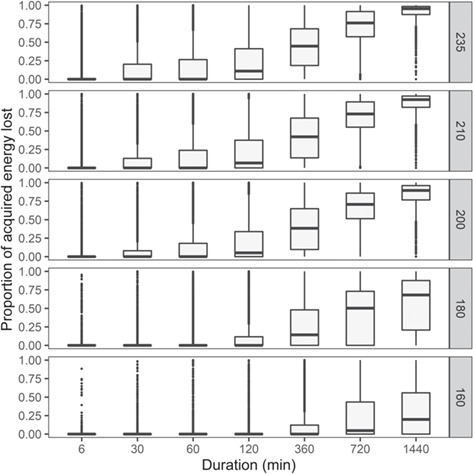
Boxplots of the predicted proportion of acquired energy lost due to disturbance, for increasing source intensity (rows) and duration. The plot shows the long tails of the corresponding distributions.

**Figure 5:**
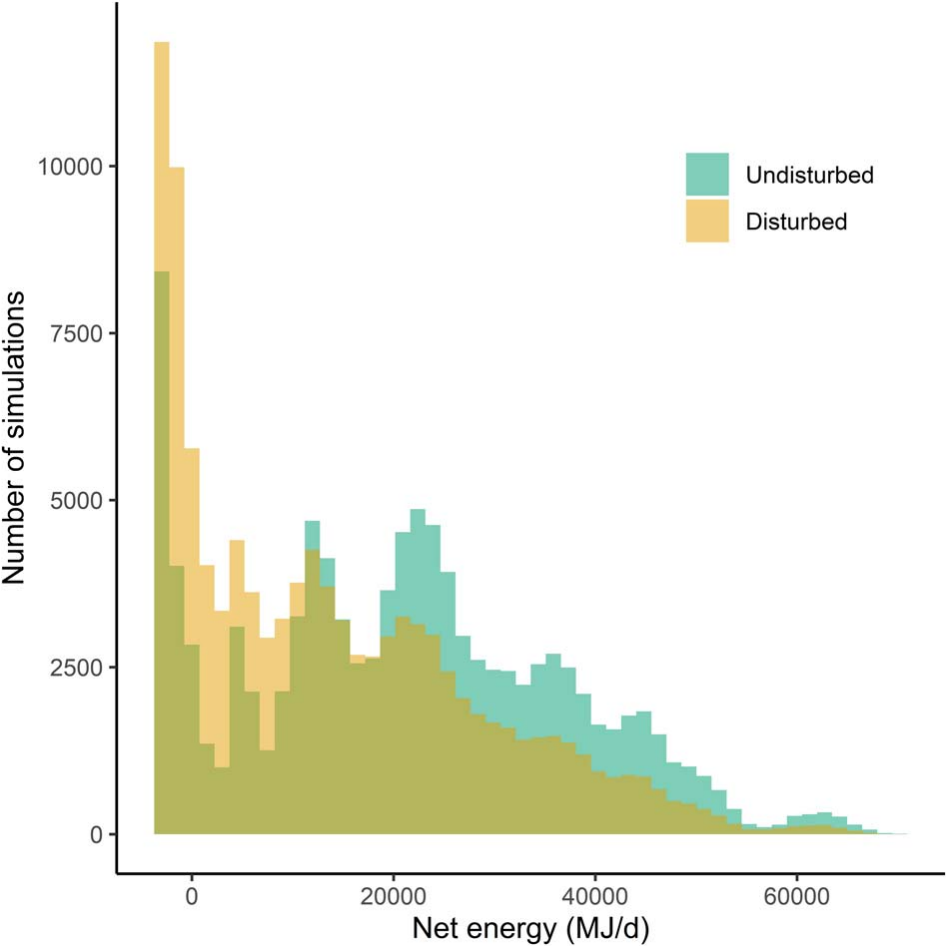
Daily net energy intake (energy acquired-energy expended) in undisturbed and disturbed conditions, assuming the discrete exposure-response function for median values under moderate response severity, the lower krill density distribution and a 22-m long individual. Net energy intake was negative when maintenance costs exceeded the net energy acquired through feeding. The third color on the graph represents the overlap between the histograms.

Given the empirical activity budgets and lunge rates, a body length of 22 m and the lower pooled krill density distribution, an individual was predicted to acquire 27 663 MJ/d (range: 0–82 593 MJ/d) and expend 6592 MJ/d (range: 2555–10 628 MJ/d), on average. When we simulated a disturbance-inducing source, the mean feeding time, number of lunges, gross energy and proportion of acquired energy that were lost as a result of exposure and any associated behavioral changes all progressively increased for increasing intensity (SL) and duration of the source (Fig. 3). However, even in scenarios involving a weak or brief source, the distribution of these variables had long tails (Fig. 4). In 51% of simulations under reference conditions, there was no change in the net energy intake for the day, either because individuals did not overlap with the source in space or time, or were exposed but did not respond. In contrast, in 49% of simulations the net energy intake decreased and in 11% of all simulations it went from positive to negative (Fig. 5). Gestation costs could increase energy expended per day by an average of 7% (range: 4–15%), while lactation costs represented a mean increase of 77% (range: 41–166%).

Mean gross energy loss was higher for larger individuals and when krill density was sampled from the upper pooled distribution. Because undisturbed energy acquisition was also higher in these cases, the proportion of daily acquisition that was lost was not affected by these variables (Supplementary Methods S3, Fig. S6). With equal SL and duration, a source positioned at the center of the location had a greater chance of overlapping with the area covered by an individual within a day, increasing overall exposure (Supplementary Methods S3, Fig. S7).

Using activity data from specific locations affected predictions: in latitude range 33.8°N--34.4°N, animals spent more time in deep-feeding state and covered smaller areas per day, leading to a mean 27% increase in time loss, which, in turn, led to a mean 45% increase in energy loss, compared to predictions from the entire dataset. Differences were less marked in latitude range 37.6°N--38.4°N due to a combination of more time not feeding or in shallow-feeding state and larger areas covered per day, resulting in a mean 7% higher time loss and 5% higher energy loss. Similarly, predictions using only activity data from July, when individuals engaged in more deep feeding, indicated a 21% higher time loss and a 29% higher energy loss, on average. In contrast, using data from October, when feeding was reduced, led to a mean 19% lower time loss and a 27% lower energy loss.

Under reference conditions, predicted effects varied depending on what discrete ER function was used. Using the function derived from the upper confidence interval of the survival analysis for moderate response severity caused a 9% higher time and energy loss, compared to using the median. Predicted mean effects were 10% lower when median values from the curve for high response severity were used, whereas they were 1% higher when using the upper confidence interval of the high curve.

Predictions differed more substantially when the two continuous ER functions were used to assess the probability of a behavioral response. Using the continuous function for SPL, mean time and energy loss was 63% lower than predicted using the discrete ER function under reference conditions (Supplementary Methods S3, Fig. S8). Because the probability of responding at a given range from the source declined to 0 within ~ 5 km (i.e. resulting in a much smaller impacted area, irrespective of source level), mean effects using this ER function were 98% lower than predicted using the discrete ER function.

## Discussion

We have demonstrated that diverse data sources obtained on different spatial and temporal scales, including high-resolution experimental exposures to disturbance-inducing stressors, activity monitoring over multiple days or weeks, telemetry tracking and prey sampling, can be effectively integrated with a bioenergetic model to predict the effects of disturbance on an individual’s daily net energy intake. We showed that these effects were heterogeneous and depended on the context of exposure. Our approach can be used to inform population-level inference, thus bridging the gap between fine-scale, experimental studies and the assessment of the long-term consequences of disturbance.

### Daily energetic costs of disturbance: large variability and context-dependency

We found that the daily energetic costs of disturbance on individual blue whales were highly variable. Source intensity and duration were obvious drivers of such variation, but the distribution of predicted costs had long tails, indicating that consequences could be dramatic across scenarios: some simulated individuals, on some days, lost all daily energy acquisition, even under brief (e.g. 6–30 min) or weak (e.g. 160–180 dB re 1 μPa source level) disturbance events (Fig. 4). In approximately 50% of simulations, net energy intake decreased and in 11% of simulations it became negative, indicating an individual would not be able to cover energy expenditure with the energy acquired on that day. In contrast, in the other 50% of simulations, no change in daily net energy intake was predicted, because feeding whales either were not exposed to the source or did not respond. The variability in predicted costs for animals that were disturbed resulted from the large behavioral variation recorded in the tag data, i.e. the differences in activity budget, lunging rates and ranging pattern among individuals and days. Observed behavioral patterns, in turn, likely reflected differences in the prey patches targeted by tagged animals (Goldbogen *et al.,* 2015; Hazen *et al.,* 2015). Similarly, large variability also emerged when activity data were restricted to a specific location or month: more intense feeding activity, concentrated in smaller areas, as observed in southern California and in summer, resulted in considerably higher predicted costs (up to 45% greater energy loss) (Pirotta *et al.,* 2018b, 2019). Reproductive costs, especially during the lactation phase (ranging approximately between December and July for this population), would exacerbate the effect of disturbance on the daily energy intake (Gittleman and Thompson, 1988).

Across taxa, including in pinnipeds (Boyd, 1999; Williams *et al.,* 2007; Dalton *et al.,* 2015; Russell *et al.,* 2015), other mammals (Hamel and Côté, 2008; Studd *et al.,* 2020) and birds (Vézina *et al.,* 2006; Louzao *et al.,* 2014) the time allocated to foraging and the resulting activity and energy budgets, is known to be affected by resource availability, seasonality, body condition and reproductive state. In parallel, movement behavior and home range size also vary within and among populations as a function of resource abundance and season, for example in ungulates (Morellet *et al.,* 2013). The large variability in daily behavioral patterns of baleen whales could emerge from their foraging strategy, whereby the high costs associated with lunge feeding must be balanced with large intake from dense, but patchily distributed prey (Goldbogen *et al.,* 2019).

The marked differences among the predicted effects of disturbance on individual blue whales underline the importance of a spatially and temporally explicit evaluation of these effects. Marine spatial planning can be used to minimize the overlap of disturbance-inducing activities with key areas or times for foraging (Foley *et al.,* 2010). More generally, our results reinforce the idea that context modulates the predicted costs of disturbance (Gill *et al.,* 2001; Tablado and Jenni, 2017). Further data collection is therefore advised to fully characterize movement, foraging patterns and prey availability and density across the population’s range, particularly around the under-sampled geographical extremes and in winter (Table 2).

**Table 2:** Knowledge gaps and associated data requirements

Knowledge gap	Data requirements	Study component highlighting the gap
Variation in whale behavior	Activity budgets, feeding rates and daily ranging patterns across latitudes and seasons	Table 1; Fig. 4
Variation in prey	Prey density measurements in different latitudes, seasons, and depth strata; prey length distributions; prey energy density	Supplementary Methods S3, Fig. S6; prey density and energy content affect an individual’s long-term energy budget and ability to compensate for predicted daily energy loss; depth-dependent densities would change predicted costs of disturbance when shallow and deep feeding
Whale sensory ecology	Identification of cues used to locate patches and assess their quality	Simulated responses require information on how quickly and efficiently an individual can resume foraging after disturbance
Context-dependency in probability of response	More CEEs for combinations of contextual variables (e.g. exposures at larger distances and lower received levels, targeting animals in different states or feeding on different prey densities)	Figs 1, 3; Supplementary Methods S3, Fig. S8
Realistic exposure scenarios	Noise propagation modelling and characterization of disturbance-inducing sources	Fig. 3; source intensity and noise propagation determine an individual’s exposure rate and level
Pathways for adverse effects	Measurement of physiological responses to disturbance (e.g. stress hormones)	Simulations only included behavioral responses; the effects of prolonged exposure were only additive

The influence of exposure context on response probability (e.g. Ellison *et al.,* 2012) was directly addressed here in the derivation of state- and range-dependent ER functions: while these functions relied on several assumptions and a mechanistic understanding of whale hearing processes to remedy the paucity of empirical data, they allowed these contextual variables to be explicitly incorporated in the simulation of responses (Goldbogen *et al.,* 2013; DeRuiter *et al.,* 2017; Southall *et al.,* 2019a). These functions were explicitly precautionary; for example, we assumed that animals exposed to a range of RL responded with a probability corresponding to the higher extreme of the range (thus inflating the effects of disturbance). Nonetheless, predicted costs were notably higher compared to the continuous functions. The continuous function for SPL predicted a higher response probability for intermediate noise levels, but lower probabilities around 160–170 dB re 1 μPa; in the discrete functions, these noise levels were assumed to cause a response in most individuals, based on considerations for levels near or exceeding those required to induce TTS. RLs above 170 dB re 1 μPa are rarely achieved in CEEs, due to permit limitations on exposure levels and difficulties in coordinating sources with mobile target animals. Thus, this discrepancy remains unresolved. Generally, while recent efforts have engaged operational sources, many CEEs will continue to rely on weaker signals compared to real sonar sources, with potential implications for the characterisation of cetacean responses, but studies based on opportunistic exposures to real Navy activities could be useful (Joyce *et al.,* 2020). The continuous function for distance, on the other hand, predicted a relatively small footprint of each disturbance event because there were few experimental exposures beyond 5 km. The lower SL used in the CEEs and the small number of distant exposures thus impose caution on the interpretation of this function.

Krill density distribution and whale body size affected gross energetic loss, but had little influence on the proportional costs at a daily scale, which were mostly driven by behavioral variability. However, as noted above, observed behavioral patterns depend on the characteristics of the prey patches on which tagged whales were feeding (Goldbogen *et al.,* 2015; Hazen *et al.,* 2015); therefore, prey availability and abundance likely played an indirect role. Moreover, patch characteristics are known to vary with depth, with resulting consequences for whale feeding strategies (Goldbogen *et al.,* 2015; Hazen *et al.,* 2015). Unfortunately, we were unable to explore the effects of this variability on the predicted costs because of sampling limitations, but future data collection should address this critical knowledge gap (Table 2). Krill density and whale size can also change the probability of animals responding to the source (Friedlaender *et al.,* 2016; Tablado and Jenni, 2017). More broadly, for long-lived species with large energy storage and fasting abilities, like the blue whale, it is the energy budget at a longer time scale that ultimately affects survival and reproduction (Pirotta *et al.,* 2019). In this sense, the variability of krill density at broader spatio-temporal scales, the number of days without any feeding and the energy storage capacity of an individual whale are critical in determining compensation after disturbance, the ability to maintain a positive energy intake over longer periods and the accumulation of sufficient reserves during the feeding season (Table 2). For example, long-term consequences will depend on whether disturbance occurs in a year where resource availability is favorable or unfavorable across the population’s range and on whether an individual is in good or bad nutritional status when it arrives on the feeding grounds (Hin *et al.,* 2019; Pirotta *et al.,* 2019).

### Data integration and the population consequences of disturbance

Understanding the long-term consequences of behavioral disruption is challenging, because extensive knowledge of baseline behavior and of the characteristics of the environment is required (Pirotta *et al.,* 2018a). In this study, the integration of multiple data sources allowed translating observed behavioral changes into a potential loss of foraging time and associated energy, which will provide a common metric to evaluate the implications of these short-term responses for individual fitness using models for the population consequences of disturbance. This reinforces the results of previous work on other marine mammal (Noren *et al.,* 2016; Farmer *et al.,* 2018; Guilpin *et al.,* 2020), mammal (Bradshaw *et al.,* 1998; Houston *et al.,* 2012) and bird species (West *et al.,* 2002; Masden *et al.,* 2010). Importantly, we were able to partially reconcile the mismatch between the scale of data collection (detailed individual movement and diving behavior, collected in specific locations and times, at extremely high resolution) and the scale of a corresponding model (Pirotta *et al.,* 2019) for population-level effects (dealing with the population, modelled across the entire year and range, at daily temporal resolution and in large spatial units), thereby condensing the data into a compatible input for the model. Given the large individual, temporal and spatial variability in the predicted energetic costs of disturbance, our results underline that modelling population consequences will require sampling from the distribution of costs, rather than using mean predictions. Ignoring this heterogeneity would be misleading, since few individuals incur average costs and some may incur much higher ones (Biggs *et al.,* 2009).

Some simplifications to the simulation approach were required in order to integrate data collected at different scales and resolutions. For example, our simple propagation model does not capture the effects of specific bathymetry and oceanography on noise propagation (Au and Hastings, 2008). Ideally, we would have used information on the realized footprints of real sonar sources in realistic training operations (Table 2), but such data are not generally available due to security restrictions. Other simplifying choices were made with regard to individual movements: a realistic movement model derived from telemetry data could be used instead (Donovan *et al.,* 2017), but fast computation will remain an important feature for real-world applications of any simulation exercise. Moreover, we used the intervals between foraging bouts to represent the time to find a new patch, similarly to Boyd (1996): in undisturbed conditions, these gaps in feeding activity probably arise from patch depletion or changes in the oceanographic processes supporting krill aggregations (Cade *et al.,* In press; Santora *et al.,* 2017). However, blue whales appear unlikely to completely leave a foraging area when disturbed, often returning to foraging rapidly following the abatement of disturbance (Southall *et al.,* 2019a). A better empirical characterization of the cues used to locate foraging patches, the distribution of patches in the environment and the factors affecting the severity of behavioral responses is therefore needed to inform this component of the simulations (Table 2). In general, we have followed the precautionary principle when addressing these limitations, which has likely led to overestimating the effects of disturbance.

The use of discrete, state- and range-specific ER functions relied on several assumptions. Future CEEs should be designed to quantitatively assess the influence of these, and other, contextual factors (Table 2), given the strong influence they had on our results (Harris *et al.,* 2018). For example, it will be important to run experiments involving multiple exposures and at larger ranges from the source (>5 km). However, the sample size required to ensure sufficient power to test complex combinations of conditions and the associated high costs of these operations are limiting factors (National Academies, 2017; Harris *et al.,* 2018). An individual’s ability or willingness to change its behavior may also depend on when exposure occurs within the dive cycle and any associated physiological constraint. Importantly, the probability of responding to a repeated or prolonged disturbance source could change over time, as a result of sensitization or habituation. This would be more accurately represented by an ER function for the aggregate exposure to noise (National Academies, 2017). Similarly, some empirical evidence suggests that animals may be responding to the total noise energy they receive, rather than the amplitude of any one stimulus (Isojunno *et al.,* 2020). Uncertainty around source characteristics and propagation, and the spatio-temporal scale of animal movements imposed by the model, made it unfeasible to use sound energy in this study. Finally, we have focused on behavioral responses, but increases in stress levels and other physiological effects of disturbance could also affect an individual’s health status on the long-term (National Academies, 2017; Pirotta *et al.,* 2018a).

In conclusion, by building on a knowledge base accrued over the last two decades across numerous empirical and analytical studies, this work demonstrates both the strengths and challenges of combining heterogeneous sources of information. These ongoing efforts will continue to collectively advance our mechanistic understanding of the behavioral and physiological pathways that underpin animals’ responses, health and life history. Ultimately, this will contribute to robust assessments of the population-level effects of anthropogenic disturbance at spatio-temporal and ecological scales that are relevant to management and conservation.

## Supplementary Material

Pirotta_et_al_Supplementary_data_revised_coaa137
