# Supplementary material for: Context-dependent variability in the predicted daily energetic costs of disturbance for blue whales
Source: Conserv Physiol. 2021 Jan 16;9(1):coaa137. doi: 10.1093/conphys/coaa137 (PMC7816799; doi:10.1093/conphys/coaa137)
Supplement: Pirotta_et_al_Supplementary_data_revised_coaa137 [file pirotta_et_al_supplementary_data_revised_coaa137.docx]

**Supplementary data**

**Supplementary Methods S1. Multi-day tag data**

*Data collection*

Tag deployments were conducted from small 6-7 m Rigid Hull Inflatable Boats (RHIBs) equipped with a bow pulpit for a tagger to stand and use a 3-4 m pole to attach tags. Tags were attached with 3-4 stainless steel darts 4-6 cm long equipped with 1-2 rows of petals (Szesciorka *et al.*, 2016; Calambokidis *et al.*, 2019). Tags were recovered after they detached from the animal and floated to the surface with the aid of an Argos satellite transmitter, which provided rough position as well as when tags had detached from the whales (based on the number and quality of positions), and a VHF transmitter that was used to localize on tags with a direction antenna.

For this study, we used 27 deployments that had at least 20 continuous hours of dive data along with high quality positions from an onboard GPS (Table S1). Tags used in this study consisted of two primary tag designs: 1) Wildlife Computers TDR10-F tag (n = 21), recording depth, temperature, light-level, Fastloc® GPS, and, in most configurations, 3D accelerometry; this tag was modified to take a plate with darts and a satellite transmitter. 2) Acousonde acoustic tag (n = 6), which is a miniature, self-contained, autonomous acoustic/ultrasonic recorder, incorporating hydrophones as well as depth, altitude and orientation sensors, digital recording electronics, data storage, and battery with modified attachment plate with darts, satellite transmitter, Sirtrack FastGPS, and custom-made syntactic floatation.

*Data processing*

Raw tag data was processed using custom MATLAB (Mathworks, version 2017b) scripts following Cade *et al.* (2016). All tags were equipped with depth and GPS sensors. Raw GPS location data were filtered for unrealistic whale speeds (> 6 m/s) using the ArgosFilter package (Version 0.62) in R (Version 3.5.1). For tags with accelerometer sensors, the animal’s pitch, roll and speed were calculated (Johnson and Tyack, 2003; Cade *et al.*, 2018).

Individual blue whale foraging events, or lunges, were identified manually by an expert analyst (Cade *et al.*, 2016). Our main sample of TDR-10-F tag deployments included an earlier model providing lower resolution data (1 Hz sampling of depth only, n = 10) and a more recent model (n = 11) with 32 Hz sampling of pressure and accelerometry. For high-resolution tag data, lunges were identified using stereotypical kinematic signatures from multiple sensors (e.g. the animal’s depth, pitch, roll and speed) (Cade *et al.*, 2016). For low-resolution tag data without accelerometry, lunges were identified using a combination of dive behavior, such as the occurrence of vertical excursions, as well as the stereotypy of those behaviors in space and time (i.e. repeated vertical excursions at similar depths). For these deployments, lunges were identified at the peak in vertical velocity for each excursion.

Because of the more limited data from the original 10 deployments of the TDR-10-F tag, we tested how the reduced resolution impacted some of the metrics used in this study by conducting a comparison of the results from the higher resolution tags to the same set of data down-sampled. Specifically, we down-sampled a subset of high-resolution deployments (n = 5; Table S2) to match the resolution of the depth-only tags (e.g. 1 Hz depth and vertical velocity) and used the same criteria (i.e. vertical excursions, stereotypy) to identify lunges.

Table S1. List of the 27 multi-day tag deployments used in the analysis (see text for details on the three types). Total hours for all tag types is 5,289 h.

| **Deployment ID** | **Deployment region** | **Tag model** | **Sensors** | **Duration (h)** |
| --- | --- | --- | --- | --- |
| Bm140719-TDR5 | Central California | TDR10-F | Depth (1Hz), GPS | 75.36 |
| Bm140825-TDR5 | Southern California Bight | TDR10-F | Depth (1Hz), GPS | 115.2 |
| Bm140825-TDR6 | Southern California Bight | TDR10-F | Depth (1Hz), GPS | 308.4 |
| Bm140827-TDR4 | Southern California Bight | TDR10-F | Depth (1Hz), GPS | 393.12 |
| Bm150818-TDR8 | Southern California Bight | TDR10-F | Depth (1Hz), GPS | 259.2 |
| Bm150819-TDR5* | Southern California Bight | TDR10-F | Depth (1Hz), GPS | 51.6 |
| Bm151016-TDR5 | Southern California Bight | TDR10-F | Depth (1Hz), GPS | 38.16 |
| Bm151016-TDR7 | Southern California Bight | TDR10-F | Depth (1Hz), GPS | 392.4 |
| Bm160523-A20 | Central California | Acousonde 3B with Sirtrack FastGPS | Depth (10Hz), GPS, Acc (100Hz), Mag (10Hz) | 97.68 |
| Bm160523-TDR6 | Central California | TDR10-F | Depth (1Hz), GPS | 186.72 |
| Bm160716-A20 | Southern California Bight | Acousonde 3B with Sirtrack FastGPS | Depth (10Hz), GPS, Acc (100Hz), Mag (10Hz) | 92.64 |
| Bm160717-A21 | Southern California Bight | Acousonde 3B with Sirtrack FastGPS | Depth (10Hz), GPS, Acc (100Hz), Mag (10Hz) | 102.48 |
| Bm160817-A21* | Southern California Bight | Acousonde 3B with Sirtrack FastGPS | Depth (10Hz), GPS, Acc (100Hz), Mag (10Hz) | 45.84 |
| Bm160918-A08* | Southern California Bight | Acousonde 3B with Sirtrack FastGPS | Depth (10Hz), GPS, Acc (100Hz), Mag (10Hz) | 98.88 |
| Bm160918-A21* | Southern California Bight | Acousonde 3B with Sirtrack FastGPS | Depth (10Hz), GPS, Acc (100Hz), Mag (10Hz) | 102.48 |
| Bm160926-TDR7 | Central California | TDR10-F | Depth (1Hz), GPS | 22.56 |
| Bm170622-TDR12 | Southern California Bight | TDR10-FX | Depth (32Hz), GPS, Acc (32 Hz) | 425.28 |
| Bm170622-TDR13 | Southern California Bight | TDR10-FX | Depth (32Hz), GPS, Acc (32 Hz) | 117.84 |
| Bm170706-TDR11 | Southern California Bight | TDR10-FX | Depth (32Hz), GPS, Acc (32 Hz) | 66.72 |
| Bm170925-TDR12 | Southern California Bight | TDR10-FX | Depth (32Hz), GPS, Acc (32 Hz) | 97.92 |
| Bm170926-TDR14 | Southern California Bight | TDR10-FX | Depth (32Hz), GPS, Acc (32 Hz) | 103.44 |
| Bm181021-TDR11 | Central California | TDR10-FX | Depth (32Hz), GPS, Acc (32 Hz) | 768.48 |
| Bm181021-TDR14 | Central California | TDR10-FX | Depth (32Hz), GPS, Acc (32 Hz) | 299.04 |
| Bm190709-TDR14 | Central California | TDR10-FX | Depth (32Hz), GPS, Acc (32 Hz) | 191.76 |
| Bm190710-TDR15 | Central California | TDR10-FX | Depth (32Hz), GPS, Acc (32 Hz) | 325.2 |
| Bm190916-TDR14 | Central California | TDR10-FX | Depth (32Hz), GPS, Acc (32 Hz) | 440.16 |
| Bm190916-TDR15 | Central California | TDR10-FX | Depth (32Hz), GPS, Acc (32 Hz) | 71.04 |

*Note: these individuals are known to have been incidentally exposed to Navy sonar.

Table S2. Details on the five high-resolution tag deployments from Table S1 that were used for comparison to a down-sampled version, mimicking the 1-Hz depth-only information available from the low-resolution tags. The table shows the false positive rate (hours with lunges after down-sampling, but not with full data) and false negative rate (hours with lunges not identified after down-sampling, but that had lunges with the full data), as well as some of the differences in lunge count and lunge depth.

| **Deployment ID** | **Total Hours** | **False Positives (h)** | **False Positives (%)** | **False Negatives (h)** | **False Negatives (%)** | **Mean lunge count** | **Mean lunge count difference** | **Mean lunge depth (m)** | **Mean lunge depth difference** |
| --- | --- | --- | --- | --- | --- | --- | --- | --- | --- |
| Bm160523-A20 | 98 | 3 | 3% | 7 | 7% | 15 | -2.4 | 29.0 | -0.6 |
| Bm160716-A20 | 92 | 2 | 2% | 0 | 0% | 24.8 | -1.7 | 161.3 | 1.3 |
| Bm170622-TDR12 | 426 | 15 | 4% | 35 | 8% | 20.8 | -0.1 | 159.2 | 0.9 |
| Bm170925-TDR12 | 98 | 3 | 3% | 3 | 3% | 16.9 | 2.1 | 227.8 | 0.1 |
| Bm170926-TDR14 | 104 | 1 | 1% | 4 | 4% | 21.4 | 7.3 | 223.1 | 3.4 |
| **Overall Mean** |  |  | **3%** |  | **4%** | **19.8** | **1.1** | **160.1** | **1** |

For the five high-resolution tags we down-sampled, we found good agreement between the high- and low-resolution measurement of key daily metrics (hours per day foraging, hourly lunge rate per day, and hourly lunge rate per day only when foraging; Fig. S1). Outlying values often represented multiple days from the same tag. Overall, the hours of false positives (hours with lunges after down-sampling, but not with full data) were lower than the false negatives (hours with lunges not identified after down-sampling, but that had lunges with the full data), but each averaged < 5% among the five tags. Similarly, differences in lunge count and lunge depth between the high-resolution and down-sampled data were small and in opposite directions for different tags, i.e. they were not biased in a consistent way. Moreover, differences were very small for four of the five tags, with most of the inconsistencies coming from one tag (Fig. S2).


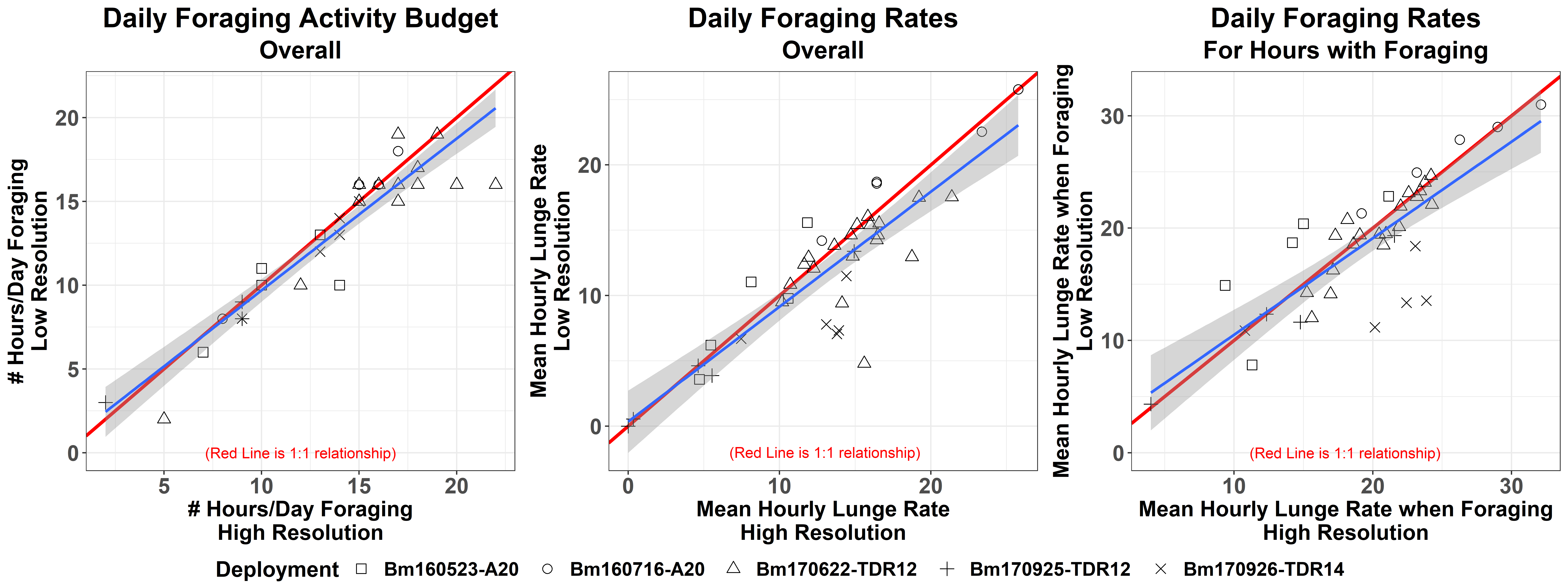


Figure S1. Daily metrics derived from high-resolution and down-sampled (low-resolution) data: a) hours per day foraging, b) hourly lunge rate per day, and 3) hourly lunge rate per day only when foraging. Red line shows 1:1 agreement between the measurements and blue line shows observed regression. Symbol type shows different days of the same tag.


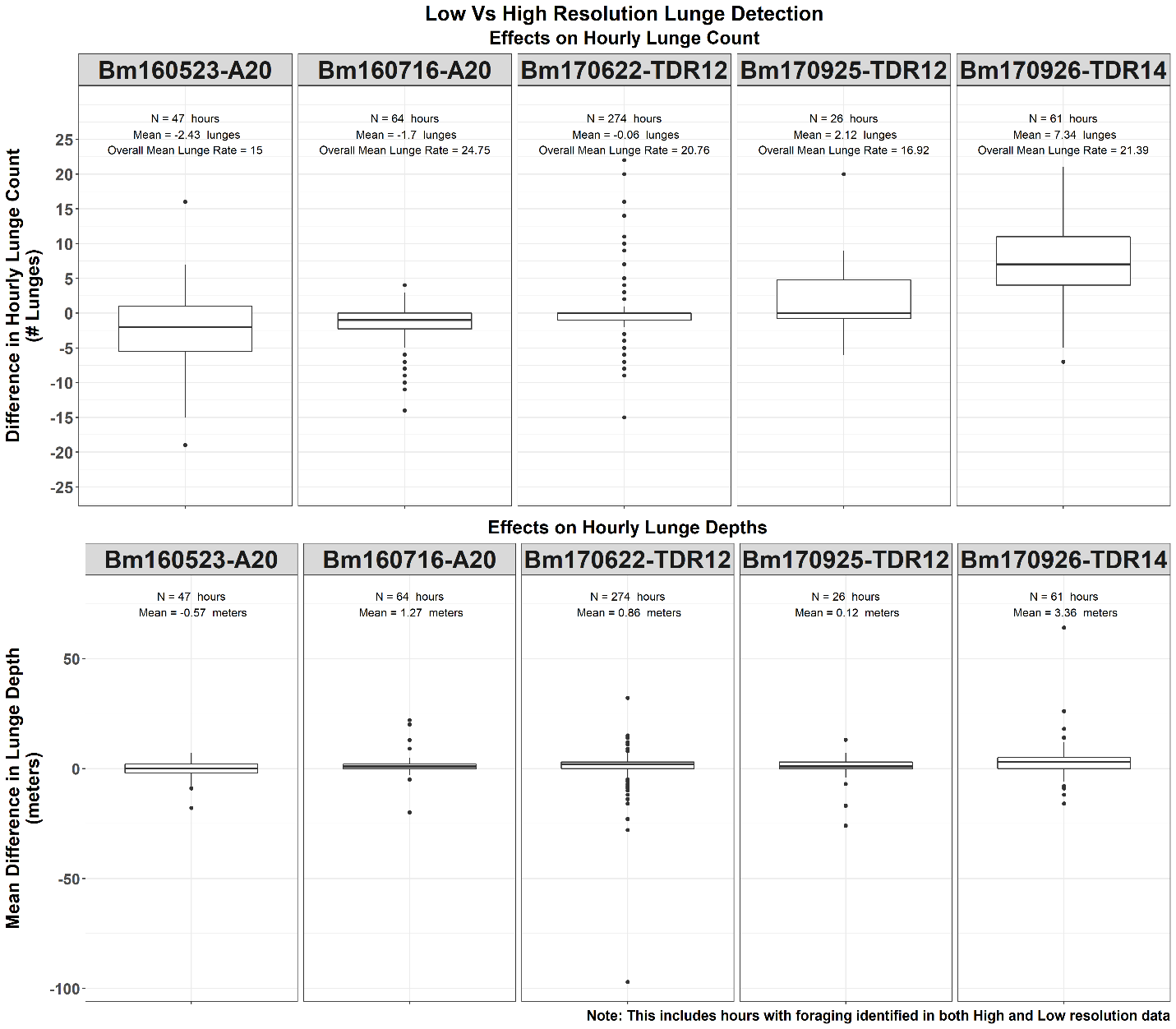


Figure S2. Differences in hourly lunge count and lunge depth between high-resolution and down-sampled (low-resolution) tags, plotted for each of the five tags in the subset.

The lunges that were missed in the down-sampled data mostly consisted of hours with shallow lunges at night (Fig. S3). These were mostly from 4 of 19 nights from a single tagged individual that engaged in rare night feeding. These misclassifications mostly reflected the greater challenge of correctly classifying shallow lunges without accelerometry data.


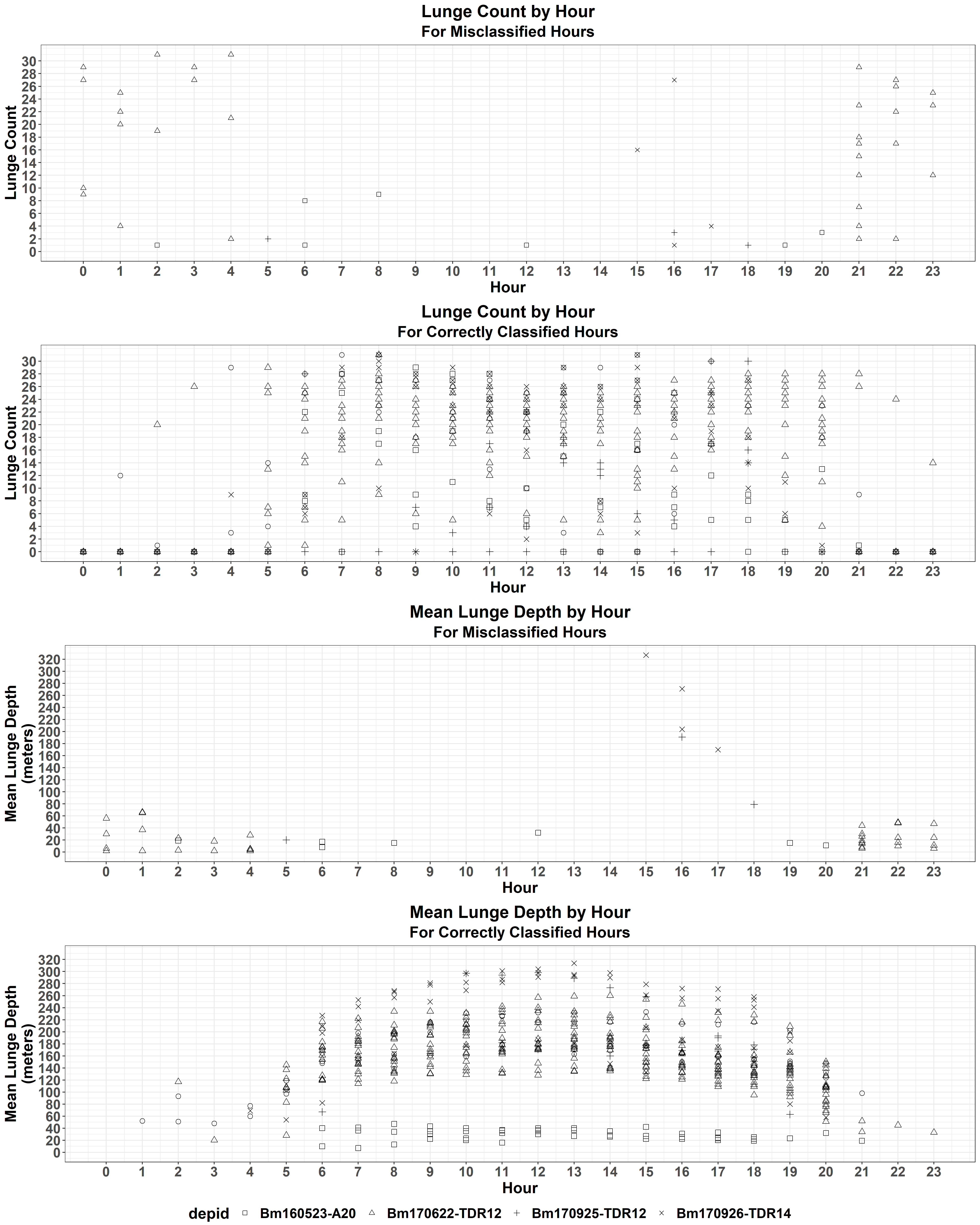


Figure S3. Distribution by hour of day for lunge count (top) and mean lunge depth (bottom), for hours where the high resolution detected lunging activity but the low resolution did not (above) and where they both had lunging (below).

We linearly interpolated whale tracks to obtain hourly locations using package adehabitatLT version 0.3.24 for R (Calenge, 2006). Given the broad spatial resolution used for the analysis (100 km x 100 km locations; see main text), linear interpolation was deemed sufficient. To avoid extrapolation over long gaps in the location data, tracks were split into separate segments whenever consecutive locations were separated by more than 10 hours. Hourly locations were then paired with hourly summaries of diving behavior.

**Supplementary Methods S2. Development of exposure-response probability functions**

*Discrete, context-relevant exposure-response functions*

There are three categories of underlying assumptions and observations from the existing literature inherent in the derivations of the deliberately coarse, context-dependent functions we used in this study. First, there is increasing scientific support for context-dependency in marine mammal behavioral response probability. Ellison *et al.* (2012) proposed a bi-phasic relationship whereby response probability to low to moderate level noise exposure is dependent on key contextual factors (e.g., spatial proximity, relative orientation, environmental factors), while high level exposures (near and approaching levels of auditory fatigue, or temporary threshold shift (TTS)) are more likely to follow linear exposure-response relationships. Frankel *et al.* (2016) and Ellison *et al.* (2018) provide further characterization and parameterization of context-dependencies in generalized behavioral response probability.

Second, there is empirical support for context-dependent behavior and behavioral responses in blue whales. Friedlaender *et al.* (2014, 2017) provide evidence of different kinds of spatial and environmental context dependency in baseline (undisturbed) blue whale behavior. Within the behavioral response study in Southern California (SOCAL-BRS), multiple analyses of blue whales exposed to mid-frequency active sonar (MFAS) and pseudo-random noise (PRN) have demonstrated context-dependency in response in different ways. Analyses across individuals clearly demonstrated stronger behavioral responses to MFAS and PRN for animals in deep-diving behavioral state (Goldbogen *et al.*, 2013; DeRuiter *et al.*, 2017). Friedlaender *et al.* (2016) further demonstrated the importance of the environmental context of prey distribution as a mediating factor in blue whale behavior and responses to disturbance. Finally, Southall *et al.* (2019a) applied both qualitative and quantitative metrics within individuals to evaluate not only the probability of response, but also the exposure conditions (including exposure received level) at which responses did or did not occur. This provides the most readily applicable data with which to develop exposure-response functions that explicitly consider key aspect of contextual responses. Southall *et al.* (2019a) used recurrent event survival analysis to determine relationship between exposure dose (cSEL) and response probability for different response severity, individual behavioral state, and source-animal range.

Third, given limitations in the underlying empirical data and the relative spatial and temporal scales being considered, a deliberately coarse probability function is justified. While even more limited data sets than those available for blue whale response probability to sonar have been used to derive continuous and explicit probability functions (see continuous exposure-response functions in this study, for reference), a different approach is taken here. The presumption behind smooth response probability functions is that a given increase in exposure level equals a corresponding increase in response probability, often with resolutions to fractions of a dB or percentage. For blue whales, certain key variables, such as the physical range over which exposures occur, were not systematically and experimentally tested. Further, while the full blue whale data set analyzed by Southall *et al.* (2019a) exceeds 40 whales, there are limited samples in certain behavioral states (e.g., non-feeding, surface feeding), meaning they necessarily need to be combined.

In light of the above observations, a deliberately coarse approach was taken in this study to derive context-dependent response functions. The empirical data from the SOCAL-BRS blue whale studies was applied, where available, to identify response probabilities of low, moderate, and high response probability, with additional data on ambient noise, hearing and auditory fatigue used to define very low and very high response probabilities at the extreme lower and upper ends of response functions. A total of five response percentage levels were envisioned (1, 10, 50, 90, 99% response probabilities), using: ambient noise levels to define the lowest probability; empirical response data to determine intermediate probabilities; and available noise exposure criteria on auditory fatigue to determine the highest probabilities. In practice, the empirical response probabilities for some contextual conditions fail to reach 90% or even 50% response probability, even at the highest received levels (i.e. response curves reached an asymptote).

Four distinct contextual conditions were defined, differentiated by behavioral state (deep-feeding; non-deep-feeding, i.e. all other states) and source-whale range (near, i.e. ≤ 1 km; far, i.e. > 1 km). Given the limited available data, these were collapsed into three contexts, under the assumption that being in the most sensitive state (deep feeding) far from the source was comparable to being in another state but close to the source. The three resulting contexts were: 1) deep-feeding, near; 2) deep-feeding, far, and other states, near; and 3) other states, far (Fig. S4). Response probabilities from the available data indicate decreasing relative sensitivity from context 1 to 3. In order to reflect differences in potential magnitude of responses, probabilistic functions were determined for each of these three behavioral contexts for moderate response severity (scores 4-6), as well as high response severity (scores 7-9). Both median predictions and the values associated with upper confidence intervals were determined, to account for statistical variance in the underlying event survival analyses. This results in four total response functions for each context: 1) moderate response severity, median predictions; 2) moderate response severity, upper confidence interval of predictions; 3) high response severity, median predictions; 4) high response severity, upper confidence interval of predictions.

| **Range from source**  **Behavioral state** | *Near (≤ 1 km)* | *Far (> 1 km)* |
| --- | --- | --- |
| *Deep-feeding* | Context 1 | Context 2 |
| *Other (non-deep-feeding)* | Context 2 | Context 3 |

Figure S4. Contextual conditions resulting from the combinations of different behavioral states and ranges from the sonar source. Background color warmth indicates the expected relative sensitivity in each context.

The following data sources were used to populate each of these four exposure-response functions:

- Estimated ambient noise in MFAS band (3-4 kHz) for sea state 3 conditions based on Wenz (1962), which were used for 1% response probabilities;

- Response probabilities from Southall *et al.* (2019a) event survival response functions directly applied at 10, 50, 90% levels. Where curves reached an asymptote below these values, received levels were determined at respective probability asymptotes;

- Because all functions in Southall *et al.* (2019a) reached an asymptote below 90% response probabilities, these values were determined using estimates of effective quiet from humans exposed to various noise as 10 dB below estimates of TTS (Ward *et al.*, 1976);

- TTS onset estimates for ‘low-frequency cetaceans’ derived from Southall *et al.* (2019b).

All received level values were converted to root mean square (RMS) values in order to match Navy received level (RL) model estimates of exposure (as well as 1/3-octave ambient noise levels). RMS values were estimated by subtracting 10 dB from cSEL values, which effectively assumes 10 MFAS pings (1 s each) received at max level. Response probabilities for each of the four functions are given below, with the basis of RL estimates for each behavioral state/range context.

Exposure-Response Function 1: Moderate response severity, median predictions

Exposure-Response Function 2: moderate response severity, upper confidence intervals

******

Exposure-Response Function 3: high response severity, median predictions

******

Exposure-Response Function 4: high response severity, upper confidence intervals

*Continuous exposure-response functions*

Continuous exposure-response functions for Sound Pressure Level (SPL) and range from source were developed using the approach described in Miller *et al.* (2014). Under the approach a process component models the noise exposure threshold at which individuals are expected to show a response, including an individual random effect and the effects of previous exposure and source type (mid-frequency active sonar, MFAS, or pseudorandom noise, PRN). The importance of the two fixed effects is assessed using Gibbs Variable Selection and the model is refitted without either of the factors if their inclusion is not supported. An observation model then links modelled thresholds with observed values, accounting for measurement error.

For the first continuous exposure-response function for SPL, the model was informed by the SPL received by an animal at the time of an identified response or, for individuals that did not respond, the maximum SPL received during the experiment. For consistency with Miller *et al.* (2014), it was assumed that no animal would respond at levels ≤ 60 dB re 1 μPa and all would respond at levels ≥ 200 dB re 1 μPa. The model was fitted using JAGS run from R (package runjags version 2.0.4; Denwood, 2016), using the same priors as Miller *et al.* (2014). Markov Chain Monte Carlo (MCMC) algorithms were iterated until convergence of the parameters. We ran three parallel chains, starting at different initial values, and discarded the first 50,000 iterations as burn-in. Convergence was assessed by visually inspecting trace and density plots, and confirmed by checking that the Brooks-Gelman-Rubin diagnostic fell below 1.1 and that the Monte Carlo error was less than 5% of the sample standard deviation. We also ensured that effective sample size was greater than 400 (Lunn *et al.*, 2013). For ease of computation and storage, we thinned the chains, retaining one in every 10 iterations.

For the second continuous ER function for range from the noise source, we extracted the distance (in km) between an individual and the source at the time of an identified response or, for individuals that did not respond, the minimum distance reached during the experiment. We used the same approach described above for SPL, adapted to account for the fact that the probability of responding is expected to decline for increasing values of range from the source. Moreover, because we could not assume that all animals would respond at 0 distance from the source, we set the lower bound to an arbitrary negative value (-5 km) and estimated a probability of response ≤ 1 at range = 0 km. The upper bound was set at 20 km, which was deemed sufficiently high since no individual in Southall *et al.* (2019a) responded beyond 2.8 km. The standard deviations for the individual random effect and for within-whale variation both had a uniform prior *U*(0, 5). The fixed effects for previous exposure and source type had Normal prior *N*(0, 10), and Gibbs Variable Selection was used as in Miller *et al.* (2014). The measurement error for the observation model was set at 0.5 km, as a conservative estimate of the uncertainty on whale locations during CEEs.

**Supplementary Methods S3. Additional details of disturbance simulations**

*Noise propagation*

In the absence of information on the noise fields around sonar sources in realistic training operations, a simplistic, spherical noise propagation model used to determine the ranges (*R*) at which received levels (RL) of interest were reached, given the source level (SL). Specifically:

RL = SL – 20∙log(*R*) – α∙*R*,

where α is an absorption of 0.2 dB/km for a nominal frequency of 3 kHz (Au and Hastings, 2008).

*Individual ranging pattern*

The area covered (*A*) in the random day sampled at each replicate was taken to represent the ranging pattern of an individual on that day. We assumed this area was entirely contained within the 100 km x 100 km location used in the simulations. For simplicity, we also assumed that such area was circular, with a radius corresponding to *r* = √*A*/π, and that the animal was using it uniformly.

*Behavioral responses*

Discrete exposure functions were constructed assuming SPL levels for up to 10 sonar pings at the maximum received level, based on the maximum number of exposures within 1 dB of the highest received level observed in individual CEEs in Southall *et al.* (2019a). This would correspond to an average duration of up to 6 minutes based on the sonar duty cycles used in CEEs with both simulated and operational Navy MFAS systems (Southall *et al.*, 2019a) (B. Southall, unpublished data). Therefore, intervals of 6-minute duration were used as the unit of time in which an individual could respond when exposed to the source (that is, a total of 240 intervals per day).

For the continuous DR functions, because of how these functions were constructed, the time interval at which an individual could respond was 30 min, i.e. the typical experimental exposure duration in Southall *et al.* (2019a), resulting in 48 intervals within a day.

We conservatively assumed that a foraging individual within the area exposed to a given range of RL had a probability of responding corresponding to the probability for the upper extreme of that range. For example, given SL = 235 dB re 1 μPa, RL = 160 dB re 1 μPa at 5 km and RL = 145 dB re 1 μPa at 20 km; if the probability of responding is 0.9 at 160 dB and 0.35 at 145 dB, the probability of responding between 5 km and 20 km from the source was 0.9.

*Bioenergetic equations*

We estimated the theoretical gross energy acquired (kJ) on day *d* without disturbance, *E*(*d*), given the corresponding bioenergetics equation in Pirotta *et al.* (2018, 2019):

*E*(*d*) = $\sum_{h=1}^{t_{f}(d)} \sum_{i=1}^{\omega(h,d)} x\left( i,h,d \right)\cdot\beta\cdot\rho\cdot A,$ where:

- *t_f_*(*d*) is the number of hours spent feeding on that day (from the multi-day tag data);
- *ω*(*h,d*) is the number of lunges in each feeding hour *h* of day *d* (i.e., the lunge rate, from the multi-day tag data);
- *x*(*i,h,d*) is a value of krill density (kg/m^3^), drawn from the pooled distribution of krill densities for each lunge *i* in each feeding hour *h* of day *d*;
- *β* = 0.0011 ∙ *L*^3.56^ is the volume of water ingested in a lunge (m^3^), given individual length *L* in m (Goldbogen *et al.*, 2009);
- *ρ* = 3,800 kJ/kg is the energy density of krill in the California Current (Chenoweth, 2018);
- *A* = 0.84 is the assimilation efficiency (Goldbogen *et al.*, 2011);

The bioenergetics equations in Pirotta *et al.* (2018, 2019) were also used to estimate daily energy expenditure, given the activity budget on each day:

*C_f_*(*d*) = *R* ∙ *t_f_*(*d*) ∙ *M* ∙ 3,600 / 1,000 is the cost of feeding (kJ) on day *d*, where:

- *R* = 26.23 ∙ *L*^-0.809^ is blue whale mass-specific Rorqual Average Active Metabolic Rate (RAAMR, W/kg), given individual length *L* in m (Potvin *et al.*, 2012);
- *t_f_*(*d*) is the time spent feeding in hours on that day (from the multi-day tag data);
- *M* is the whale’s mass in kg, and is equal to 4.6 ∙ *L*^3.05^ (Lockyer, 1976);
- The multiplication by 3,600 is used to obtain J/h from W (= J/s), while the division by 1,000 is used to covert J to kJ.

*C_o_*(*d*) = *t_o_*(*d*) ∙ *O* is the cost of other activities (kJ) on day *d*, where:

- *O* = 2 ∙ *BMR* is the non-feeding metabolic rate in kJ/h (Potvin *et al.*, 2012);
- *BMR* = 4 ∙ *M*^0.75^ ∙ 3,600 / 1,000 is the hourly basal metabolic rate (kJ/h) [Kleiber (1975) in Potvin *et al.* (2012)];
- *t_o_*(*d*) = 24 – *t_f_*(*d*) is the time spent not feeding, in hours.

*N*(*d*) = *E*(*d*) – *C_f_*(*d*) – *C_o_*(*d*) is the net energy intake on day *d* in undisturbed conditions (i.e., acquired minus expended energy). Disturbance affects both the energy acquired (i.e., *E*(*d*) is reduced to account for lost energy) and the energy expended (i.e., *t_f_*(*d*) is reduced to account for lost feeding time, affecting both *C_f_*(*d*) and *C_o_*(*d*)).

*α_g_*(*g*) = Δ*w_f_*(*g*) ∙ *c_f_* + *h*(*g*) is the daily cost of gestation on day of gestation *g*, where:

- Δ*w_f_*(*g*) = *w_f_*(*g*) – *w_f_*(*g* – 1) is the change in weight of the foetus from the previous day, given the weight of the foetus on day *g,* *w_f_*(*g*) = [0.52 ∙ (*g* – 73)]^3^ / 1,000 [Huggett and Widdas (1950) in Lockyer (1981)];
- *c_f_* = 12,301 kJ/kg is the cost of pregnancy per unit of foetus weight (Lockyer, 1981, 1987, 2007);
- *h*(*g*) = *p*(*g*) ∙ (4,400 ∙ 4.184 ∙ *w_b_*^1.2^) is the heat of gestation, given *p*(*g*) = Δ*w_f_*(*g*) / *w_b_*, i.e., the proportional change in foetus weight on that day with respect to the weight at birth (*w_b_* = 2,500 kg);

The cost of gestation was calculated for an individual in the middle of pregnancy (i.e., *g* = 167).

*C_L_* = *M_max_* ∙ *e* / E is the cost of lactation (kJ) assuming an individual delivered the maximum daily amount of milk to the calf, where:

- *M_max_* = 220 kg is the maximum amount of milk delivered in a day (Oftedal, 1997);
- *e* = 17,309 kJ/kg is the energy content per unit weight of milk (Lockyer, 1981);
- *E* = 0.9 is the mammary gland efficiency [Brody (1968) in Lockyer (1981)].


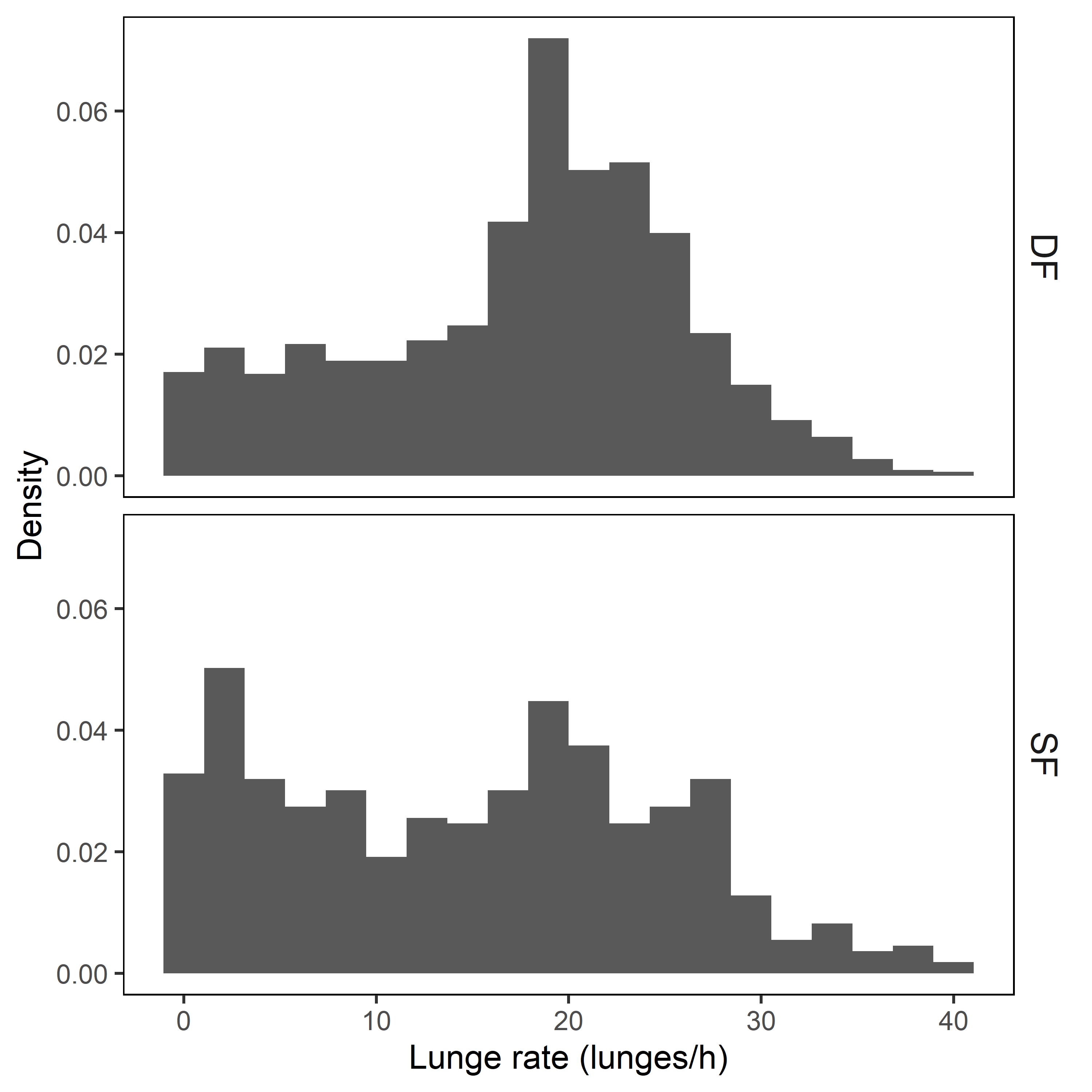


Figure S5. Distribution of hourly lunge rates by feeding state (deep feeding, DF, and shallow feeding, SF), as derived from the multi-day tag data.


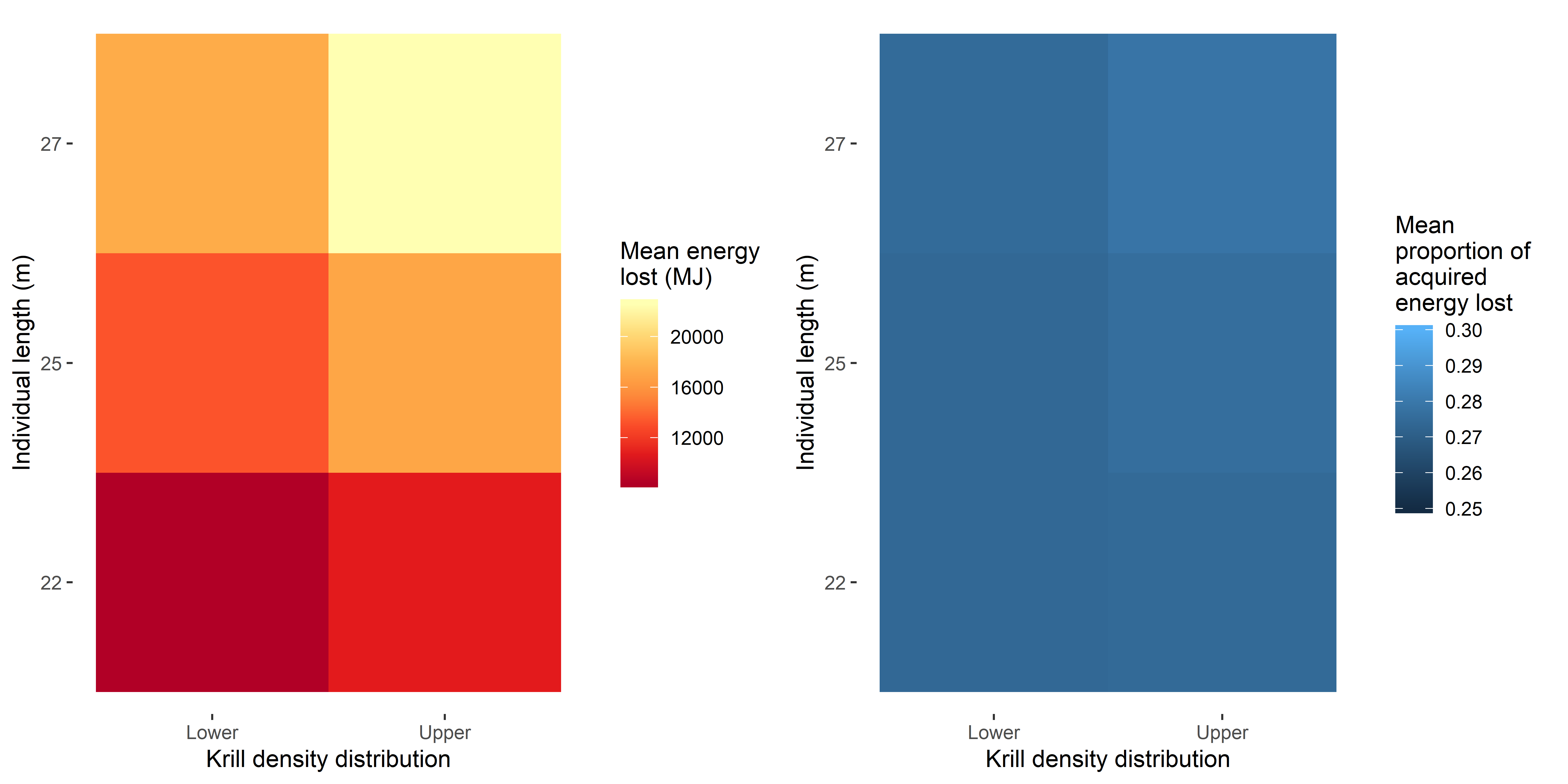


Figure S6. Predicted mean energy lost and mean proportion of acquired energy lost due to disturbance as a function of the assumed length of an individual and krill density distribution.


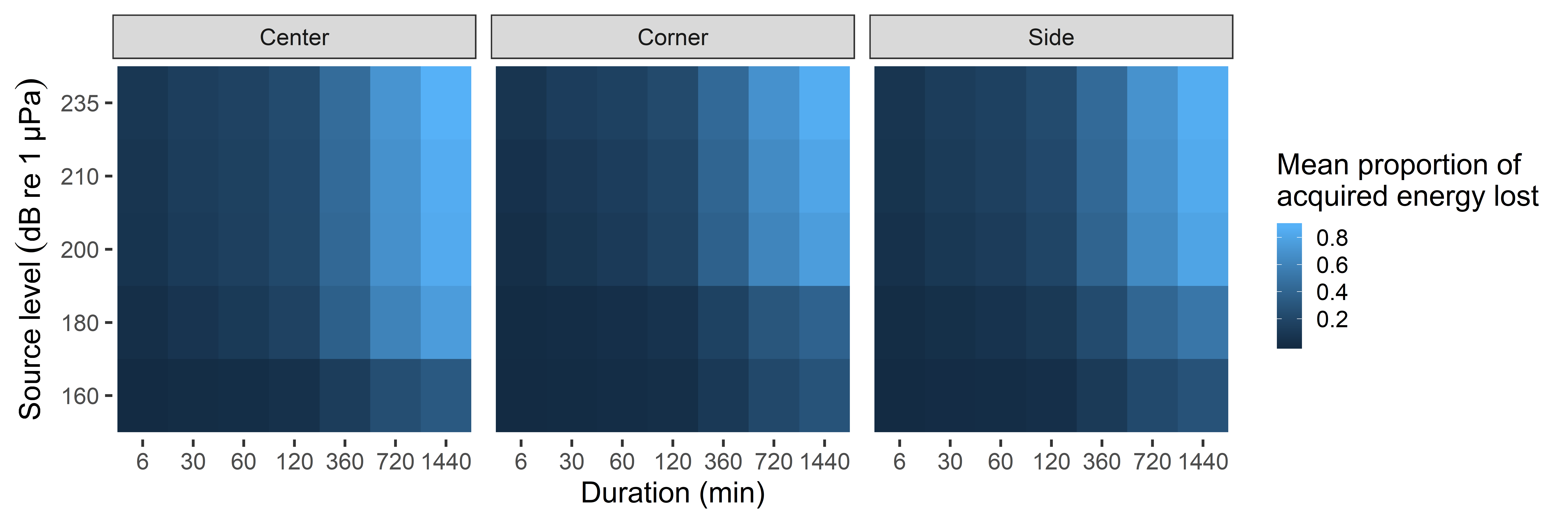


Figure S7. Predicted mean proportion of daily acquired energy lost, for increasing source intensity and duration, plotted by position of the source (at the center, in a corner or at the center of one of the sides of the 100 km x 100 km location). Predictions are plotted for reference conditions (that is, assuming the discrete exposure-response function for median values under moderate response severity, the lower krill density distribution and a 22-m long individual).


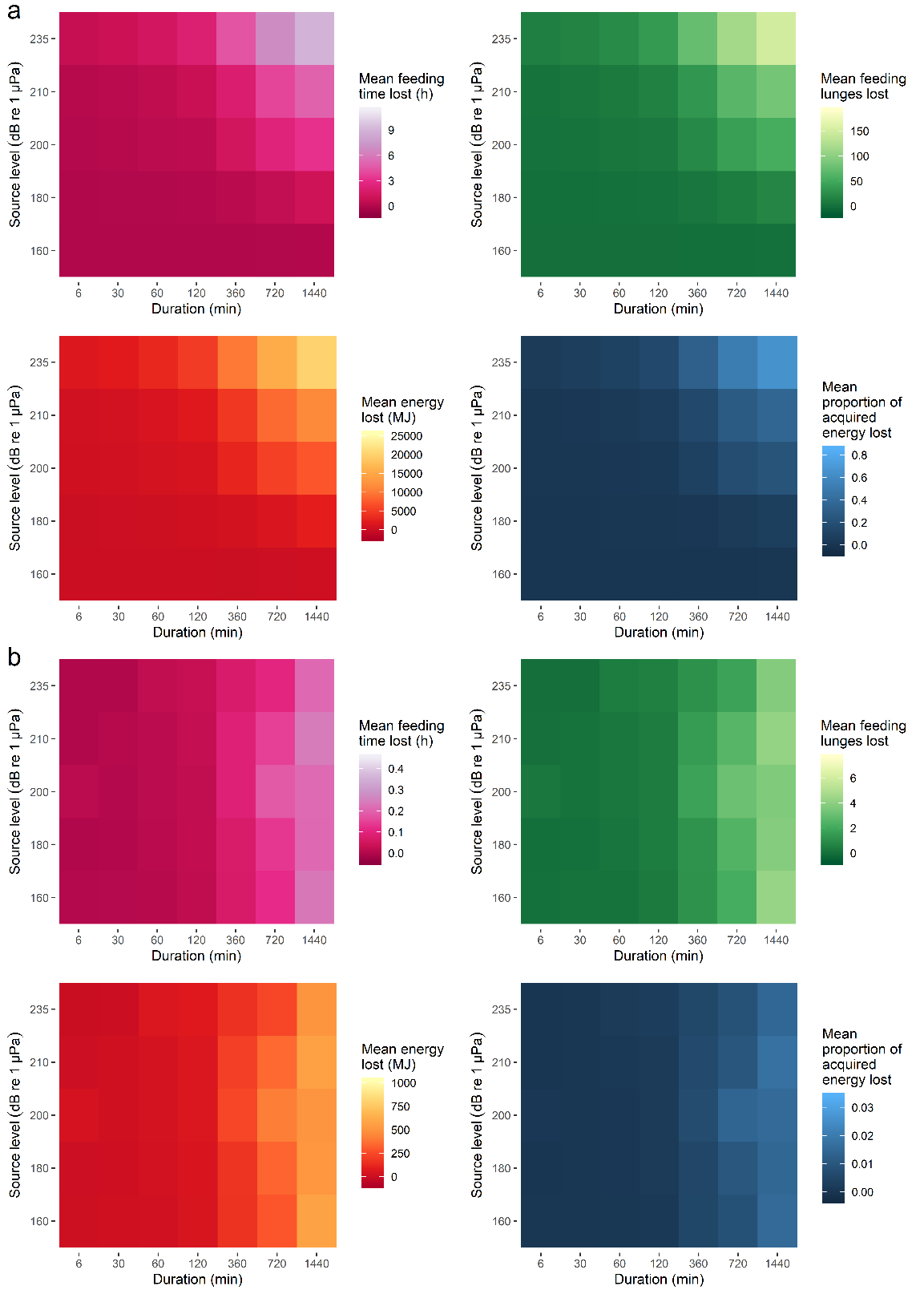


Figure S8. Predicted effects of disturbance for increasing intensity (source level) and duration of disturbance, using A) the continuous exposure-response function for received noise level and B) the continuous exposure-response response for range from source. Note that the scales in B have an upper limit corresponding to 1/25^th^ of the maximum values in A.

**Supplementary Methods S4. Table of abbreviations used in the paper**

| **Abbreviation** | **Explanation** |
| --- | --- |
| CEE | Controlled Exposure Experiment |
| ENP | Eastern North-Pacific |
| ER | Exposure-Response (function) |
| MFAS | Mid-Frequency Active Sonar |
| RL | Received Level |
| RMS | Root Mean Square |
| SPL | Sound Pressure Level |
| SL | Source Level |
| TTS | Temporary Threshold Shift |

**References cited in the Supplementary data**

Au WWL, Hastings MC (2008) Principles of Marine Bioacoustics. Springer Verlag, New York, NY.

Brody S (1968) Bioenergetics and Growth. Hafner Publishing Co., Inc., New York, NY.

Cade DE, Barr KR, Calambokidis J, Friedlaender AS, Goldbogen JA (2018) Determining forward speed from accelerometer jiggle in aquatic environments. *J Exp Biol* 221: 170449.

Cade DE, Friedlaender AS, Calambokidis J, Goldbogen JA (2016) Kinematic diversity in rorqual whale feeding mechanisms. *Curr Biol* 26: 2617–2624.

Calambokidis J, Fahlbusch JA, Szesciorka AR, Southall BL, Cade DE, Friedlaender AS, Goldbogen JA (2019) Differential vulnerability to ship strikes between day and night for blue, fin, and humpback whales based on dive and movement data from medium duration archival tags. *Front Mar Sci* 6: 543.

Calenge C (2006) The package adehabitat for the R software: a tool for the analysis of space and habitat use by animals. *Ecol Modell* 197: 516–519.

Chenoweth EM (2018) Bioenergetic and Economic Impacts of Humpback Whale Depredation at Salmon Hatchery Release Sites. University of Alaska Fairbanks.

Denwood MJ (2016) runjags: An R package providing interface utilities, model templates, parallel computing methods and additional distributions for MCMC models in JAGS. *J Stat Softw* 71: 1–25.

DeRuiter SL, Langrock R, Skirbutas T, Goldbogen JA, Chalambokidis J, Friedlaender AS, Southall BL (2017) A multivariate mixed hidden Markov model for blue whale behaviour and responses to sound exposure. *Ann Appl Stat* 11: 362–392.

Ellison WT, Southall BL, Clark CW, Frankel AS (2012) A new context-based approach to assess marine mammal behavioral responses to anthropogenic sounds. *Conserv Biol* 26: 21–28.

Ellison WT, Southall BL, Frankel AS, Vigness-Raposa K, Clark CW (2018) An acoustic scene perspective on spatial, temporal, and spectral aspects of marine mammal behavioral responses to noise. *Aquat Mamm* 44: 239–243.

Frankel AS, Ellison WT, Vigness-Raposa KJ, Giard JL, Southall BL (2016) Stochastic modeling of behavioral response to anthropogenic sounds. In: The Effects of Noise on Aquatic Life II. Springer, New York, pp 321–329.

Friedlaender AS, Goldbogen J a., Hazen EL, Calambokidis J, Southall BL (2014) Feeding performance by sympatric blue and fin whales exploiting a common prey resource. *Mar Mammal Sci*. doi:10.1111/mms.12134

Friedlaender AS, Hazen EL, Goldbogen JA, Stimpert AK, Calambokidis J, Southall BL (2016) Prey-mediated behavioral responses of feeding blue whales in controlled sound exposure experiments. *Ecol Appl* 26: 1075–1085.

Friedlaender AS, Herbert-Read JE, Hazen EL, Cade DE, Calambokidis J, Southall BL, Stimpert AK, Goldbogen JA (2017) Context-dependent lateralized feeding strategies in blue whales. *Curr Biol* 27: R1206–R1208.

Goldbogen JA, Calambokidis J, Oleson E, Potvin J, Pyenson ND, Schorr G, Shadwick RE (2011) Mechanics, hydrodynamics and energetics of blue whale lunge feeding: efficiency dependence on krill density. *J Exp Biol* 214: 131–146.

Goldbogen JA, Potvin J, Shadwick RE (2009) Skull and buccal cavity allometry increase mass-specific engulfment capacity in fin whales. *Proc R Soc B Biol Sci* 277: 861–868.

Goldbogen JA, Southall BL, DeRuiter SL, Calambokidis J, Friedlaender AS, Hazen EL, Falcone EA, Schorr GS, Douglas A, Moretti DJ, *et al.* (2013) Blue whales respond to simulated mid-frequency military sonar. *Proc R Soc London Ser B Biol Sci* 280: 20130657.

Huggett ASG, Widdas WF (1950) The relationship between mammalian foetal weight and conception age. *J Physiol* 4: 306–303.

Johnson MP, Tyack PL (2003) A digital acoustic recording tag for measuring the response of wild marine mammals to sound. *IEEE J Ocean Eng* 28: 3–12.

Kleiber M (1975) The Fire of Life. An Introduction to Animal Energetics. R.E. Kteiger Publishing Co, Huntington, NY.

Lockyer C (1976) Body weights of some species of large whales. *ICES J Mar Sci* 36: 259–273.

Lockyer C (1981) Growth and energy budgets of large baleen whales from the Southern Hemisphere. *Mamm seas (FAO Fish Ser no 5)* 3: 379–487.

Lockyer C (1987) Evaluation of the role of fat reserves in relation to the ecology of North Atlantic fin and sei whales. *Approaches to Mar Mammal Energ A C Huntley, D P Costa, G A J Worthy M A Castellini (eds) Soc Mar Mammal - Spec Publ No 1*.

Lockyer C (2007) All creatures great and smaller: a study in cetacean life history energetics. *J Mar Biol Assoc UK* 87: 1035–1045.

Lunn D, Jackson C, Best N, Thomas A, Spiegelhalter D (2013) The BUGS Book: A Practical Introduction to Bayesian Analysis. Chapman & Hall/CRC, Boca Raton, Florida, USA.

Miller PJO, Antunes RN, Wensveen PJ, Samarra FIP, Alves AC, Tyack PL, Kvadsheim PH, Kleivane L, Lam F-PA, Ainslie MA, *et al.* (2014) Dose-response relationships for the onset of avoidance of sonar by free-ranging killer whales. *J Acoust Soc Am* 135: 975.

Oftedal OT (1997) Lactation in whales and dolphins: evidence of divergence between baleen- and toothed-species. *J Mammary Gland Biol Neoplasia* 2: 205–230.

Pirotta E, Mangel M, Costa DP, Goldbogen J, Harwood J, Hin V, Irvine LM, Mate BR, McHuron EA, Palacios DM, *et al.* (2019) Anthropogenic disturbance in a changing environment: modelling lifetime reproductive success to predict the consequences of multiple stressors on a migratory population. *Oikos* 128: 1340–1357.

Pirotta E, Mangel M, Costa DP, Mate B, Goldbogen J, Palacios DM, Huckstadt L, McHuron EA, Schwarz L, New L (2018) A dynamic state model of migratory behavior and physiology to assess the consequences of environmental variation and anthropogenic disturbance on marine vertebrates. *Am Nat* 191: E40–E56.

Potvin J, Goldbogen JA, Shadwick RE (2012) Metabolic expenditures of lunge feeding rorquals across scale: implications for the evolution of filter feeding and the limits to maximum body size. *PLoS One* 7: e44854.

Southall BL, DeRuiter SL, Friedlaender A, Stimpert AK, Goldbogen JA, Hazen E, Casey C, Fregosi S, Cade DE, Allen AN, *et al.* (2019a) Behavioral responses of individual blue whales (*Balaenoptera musculus*) to mid-frequency military sonar. *J Exp Biol* 222: 190637.

Southall BL, Finneran JJ, Reichmuth C, Nachtigall PE, Ketten DR, Bowles AE, Ellison WT, Nowacek DP, Tyack PL (2019b) Marine mammal noise exposure criteria: Updated scientific recommendations for residual hearing effects. *Aquat Mamm* 45: 125–232.

Szesciorka AR, Calambokidis J, Harvey JT (2016) Testing tag attachments to increase the attachment duration of archival tags on baleen whales. *Anim Biotelemetry* 4: 18.

Ward WD, Cushing EM, Burns EM (1976) Effective quiet and moderate TTS: Implications for noise exposure standards. *J Acoust Soc Am* 59: 160–165.

Wenz GM (1962) Acoustic ambient noise in the ocean: spectra and sources. *J Acoust Soc Am* 34: 1936–1956.
